# Proceedings of the 17th Annual International INEBRIA Conference

**DOI:** 10.1186/s13722-022-00308-3

**Published:** 2022-06-29

## Oral Presentations

### O1 A learning collaborative to improve care for unhealthy alcohol use: barriers and facilitators

#### Fern McCree^1*^, Liu Junqing^1^, Jennifer Strohmeyer^1^, Ashli Barnes^1^, Danielle Rainis^1^, Serene Olin^1^, Emily Morden^1^, Natalie Omar^1^, Patricia Santora^2^, Lela McKnight-Eily^3^

##### ^1^National Committee for Quality Assurance, USA; ^2^Substance Abuse and Mental Health Services Administration, USA; ^3^Centre for Disease Control and Prevention, USA

###### Correspondence: Fern McCree (mccree@ncqa.org)

*Addiction Science & Clinical Practice* 2022,**17(Suppl 1)**: O1

**Background: **Excessive alcohol use is a leading cause of death in the U.S. Alcohol screening and brief interventions reduce unhealthy alcohol use but are underutilized in primary care. The HEDIS Unhealthy Alcohol Use Screening and Follow-Up (ASF) measure assesses unhealthy alcohol use screening among adults, and among those who screen positive, receipt of follow-up care. Reporting the measure requires use of electronic clinical data to which health plans may not have ready access. This study identified barriers and facilitators to improving reporting and performance of the ASF measure.

**Methods: **We convened a three-year (2017–2020) Quality Improvement (QI) Learning Collaborative with three health plans differing in enrollment size, geographic location, and network type. Plans engaged in QI activities (e.g. plan-do-study-act) reported HEDIS data and participated in cross-site learning meetings.

**Results: **Overall, from 2017–2019, data submitted at the health plan level showed screening rates ranged from 0 to 46% and follow-up rates from 0.4% to 79%. Screening rates increased for one plan after accessing data from one practice but decreased for two plans due to more accurate measure calculations in the last two years. Plans improved follow-up rates due to increased engagement with providers. The Collaborative revealed unique barriers and facilitators underlying performance variation.

Barriers encountered were plans limited access to clinical data, inconsistent capture of alcohol use information at practice level, reluctance of providers and patients to discuss alcohol use due to stigma, and limited brief intervention resources in primary care.

Facilitators identified were the ability to standardize data fields in provider electronic health records, establishment of data sharing agreements with large practices, education of providers, and incorporation of the ASF measure into clinical workflows.

**Conclusions: **Obtaining clinical data from providers for HEDIS ASF measure reporting was challenging. Strategic and persistent QI efforts increased data access and improved care and follow-up.

### O2 A novel computer-based avatar-delivered alcohol reduction intervention: feasibility, adaptability and acceptability among PLHIV/TB adults in Indian clinical settings

#### Nishi Suryavanshi^1,2^*, Gauri Dhumal^1^, Samyra R. Cox^2^, Shashikala Sangle^3^, Andrea Deluca^4^, Manjeet Santre^3^, Amita Gupta^2^, Geetanjali Chander^2^, Heidi Hutton^2^

##### ^1^Byramjee Jeejeebhoy Government Medical College/Johns Hopkins University Clinical Trials Unit, Jai Prakash Narayan Road, Pune, Maharashtra, India; ^2^Johns Hopkins University, School of Medicine, 600 North Wolfe Street, Baltimore, MD, USA; ^3^Byramjee Jeejeebhoy Government Medical College/Sassoon General Hospital, Jai Prakash Narayan Road, Pune, Maharashtra, India; ^4^Johns Hopkins University, Bloomberg School of Public Health, 615 North Wolfe Street, Baltimore, MD, USA

###### Correspondence: Nishi Suryavanshi (nishisuryavanshi@hotmail.com)

*Addiction Science & Clinical Practice* 2022,**17(Suppl 1)**: O2

**Background: **Hazardous alcohol use is associated with increased morbidity and mortality among persons with HIV and Tuberculosis (TB). In the US, Computer-Based Avatar-Delivered (CBAD) interventions can reduce hazardous alcohol use, are scalable, and thus, have the potential to improve outcomes among HIV/TB patients. We assessed the feasibility and acceptability of an innovative CBAD intervention for unhealthy alcohol reduction in HIV/TB clinical settings.

**Materials and methods: **In Pune, India we conducted ten in-depth interviews (IDIs) with HIV/TB patients with alcohol use disorder (AUD), two focus group discussions (FGDs) with health care providers (HCPs) from a Public hospital-based HIV clinic and one FGD with peer educators from alcoholics anonymous (AA). Participants reviewed and provided feedback on an innovative Avatar; 3-D bird, Peedy delivered intervention for AUD. Data were analyzed using structured framework analysis.

**Results: **Majority (n = 9/10) of IDI respondents were males with median age 42 (IQR; 38–45) years. HCPs of FGDs were mainly females (n = 17/22) and members of AA group were males with AUD (n = 13). Results were organized into 4 domains: Avatar Acceptability; Feedback on intervention content and time spent; Recommendations for improvement; and Feasibility in clinic settings. Overall, HIV/TB participants found Peedy to be acceptable and were comfortable honestly answering alcohol-related questions. FGD participants felt that CBAD provides systematic counselling and the intervention would be more effective with the addition of in person counseling. All participants reported the 15–20-min delivery time and the amount of information provided as appropriate. Adding visuals and an option to choose a human avatar were additional suggestions. HCPs thought CBAD could be easily implemented in clinic settings.

**Conclusions: **A CBAD alcohol reduction intervention is acceptable and feasible in Indian settings with the inclusion of in-person counseling and a human avatar to increase engagement. The findings will enhance the intervention by making culturally appropriate modifications as recommended by the participants.

Center For AIDS Research micro grant, Johns Hopkins University.

### O3 A pilot study evaluating self-reported outcomes among hepatologists attending a program for training on alcohol use disorder management

#### Jayant Mahadevan^1^, Udit Panda^1^, Daniel PR Kavati^1^, C. Likhitha^1^, N. Kubenthiran^1^, Prabhat K. Chand^1^*, Ashwani Singhal^2^, Pratima Murthy^1^, Sanjeev Arora^3^

##### ^1^Department of Psychiatry, National Institute of Mental Health and Neurosciences, Bengaluru, Karnataka, India; ^2^Department of Internal Medicine, University of South Dakota, Vermillion, South Dakota, USA; ^3^Department of Internal Medicine, University of New Mexico Health Sciences Center, Albuquerque, New Mexico, USA

###### Correspondence: Prabhat K. Chand (chand@nimhans.ac.in)

*Addiction Science & Clinical Practice* 2022,**17(Suppl 1)**: O3

**Background: **Alcohol related liver disease (ARLD) is the most common end organ complication linked to heavy alcohol use. Abstinence from alcohol is paramount in the treatment of ARLD. However, treatment for the same often does not occur in medical settings due to a lack of mental health professionals. Hepatologists are ideally placed to provide interventions for AUD, in many settings. But, a lack of specialised training in delivering interventions has been cited by many as a deterrent. Given this felt need, NIMHANS has initiated a Program for Advanced Training of Hepatologists in Alcohol Use Disorder Management (PRATHAM) using the ECHO model. 

**Methods: **The training program consists of weekly virtual tele-mentoring sessions based on the ECHO model, for a duration of 8 weeks. The focus is screening, pharmacotherapy and brief psychological interventions for AUD. Multi-point video conferencing is being used by the 'HUB' (NIMHANS) to conduct didactics, case discussions and share best practices with the 'SPOKES' (Hepatologists from USA, France, Spain, Chile and India). The program evaluation consists of a pre-post design using a survey questionnaire to document the impact of the training on knowledge, attitudes, practices and self-efficacy of the participants. 

**Results: **A total of 20 hepatologists were invited to participate in the program, of whom 18 completed the baseline evaluation. A majority (12/18) had been practicing for greater than 10 years. In general, positive attitudes towards patients with AUD were noted, with 14/18 totally agreeing that treatment was possible. A lack of self-perceived competency was observed in the areas of long-term pharmacotherapy and psychological interventions such as relapse prevention. 

**Conclusion: **This first of its kind program aims to work with hepatologists from around the world to upgrade their skills to manage patients with AUD. This will likely help them to provide comprehensive services to patients with ARLD.

### O4 Alcohol use patterns during COVID-19 pandemic in India: an exploratory online study

#### Gayatri Bhatia^1^*, Arpit Parmar^2^, Siddharth Sarkar^3^

##### ^1^National AIDS Control Organization, New Delhi, Indi; ^2^All India Institute of Medical Sciences, Bhubaneshwar, Orissa, India; ^3^Associate Professor, All India Institute of Medical Sciences, New Delhi, India

###### Correspondence: Gayatri Bhatia (gayatribhatia90@gmail.com)

*Addiction Science & Clinical Practice* 2022,**17(Suppl 1)**: O4

**Background: **The COVID 19 pandemic and the nation-wide lockdown instituted in India in March, 2020 led to ban over sales of alcohol initially which was later lifted. Combined with restriction of movement and disruption in daily routine, change in alcohol use patterns of the population was anticipated. This study aimed to assess alcohol use patterns during COVID 19 pandemic in India.

**Methods: **An online survey link enquiring about alcohol use was shared through social media employing snowball sampling techniques. Responses were gathered in the last week of May 2020 and first week of June 2020.

**Results: **The findings suggest that 65% of those participants who had consumed alcohol at least once in the previous year had continued alcohol consumption during the pandemic. More than 60% reported an increase in the money spent on alcohol consumption. While 17.5% reported problematic alcohol use, more than 30% had attempted cut-down/cessation. Help or treatment seeking was extremely low. The most common reasons for continuing alcohol use during the pandemic were stress reduction and mood upliftment. Those who continued alcohol use had several misconceptions regarding alcohol use in COVID 19 pandemic.

**Conclusion: **Lockdown in India did not result in complete cessation of alcohol use for many individuals. Use of alcohol to address negative emotional states would need to be countered to prevent emergence of alcohol use disorders.

### O5 An exploration of the feasibility and acceptability of delivering screening and brief interventions to women in prison

#### Jennifer Ferguson, Maggie Lesse

##### Teesside University, England, UK

*Addiction Science & Clinical Practice* 2022,**17(Suppl 1)**: O5

**Background: **Whilst it is well evidenced that the prevalence of alcohol misuse is high in the criminal justice system (Newbury-Birch, 2016), and it can be shown it is for women on their own also, it is important to investigate the differences between men and women, in order to tailor interventions to this specific population. More females are found to be risky drinkers when they arrive in prison (24%) compared to males (18%)(Ministry of Justice., 2018). This research aimed to approach this unmet need.

**Methods: **12 in depth qualitative interviews were completed with residents in an open prison and 6 with relevant staff and stakeholders. Thematic analysis of the transcripts was undertaken and recommendations for a future pilot study were made.

**Results: **The research highlighted the importance of using the 10 question AUDIT to establish rapport as well as its main purpose of screening. Participants discussed pragmatic issues such as follow up in this vulnerable population, timing of the intervention components and the visual aid used to guide the intervention itself. One of the main findings was the element of staff rapport within the setting. It was surprisingly a uniformed officer who was most favoured for delivery of the intervention. The findings aligned with the already evidenced pains of imprisonment (Sykes, 1958, Crewe et al., 2017).

**Conclusions: **SBI with women in an open prison setting is both feasible and acceptable provided time the results of this study are implemented in the delivery.

### O6 Application of BI module for persons with cannabis dependence (CD)

#### Mohammad A. Shah*, K. S. Sengar.

##### Department of Clinical Psychology, Ranchi Institute of Neuro-Psychiatry & Allied Sciences, Ranchi, India.

###### Correspondence: Mohammad A. Shah (shahpsych@outlook.com)

*Addiction Science & Clinical Practice* 2022,**17(Suppl 1)**: O6

**Background: **Cannabis use among people continues to rise and is at an all-time high as many states in the US and other countries lifted ban on cannabis use. With more use, more problems are expected as people develop dependence related issues. Extensive evidence shows the promising results of BI's in reducing substance abuse. Our study aims to increase healthcare access for CD by using BI module for people with CD in Indian setting.

**Methods: **The sample is composed of individuals (N = 12) having CD as diagnosed by psychiatrist with associated symptoms of depression, anxiety, poor quality of life and poor interpersonal relationships. Among these 12 individuals, 6 have been assigned to experimental group and other 6 to control group. A purposive sampling technique with pre and post test design was used. Tools used in the study include WHOQOL-BREF, DASS-21, and WCQ. The intervention was delivered to the participants in an experimental group while control group was put on treatment as usual (TAU). At 2-month follow-up participants were interviewed to understand the appropriateness of the intervention. The impact was studied in both groups through changes in consumption of cannabis and associated symptoms.

**Results: **Initial results were promising as patient's symptoms related to depression and anxiety and adjustment to the surroundings improved drastically. The patients (83.33%) were ready to discuss their relationship problems with family members as they found themselves educated about issues within the family. Almost sixty-six (66.66) percent of patients improved symptomatically after attending BI module. Further findings will be ready for presentation at the conference.

**Conclusion: **These findings provided important insights regarding interventions for people with CD. More focus on family-based intervention is needed as all participants had conflict with family members. Given these findings, further research is needed with large group of participants regarding the effectiveness of BI's in CD.

### O7 Are nurses in government hospitals of a metro city equipped with knowledge and skill to manage psychoactive substance use in their patients?

#### K. Sugavanaselvi*, Prasanthi Nattala, Pratima Murthy, B. A. Arvind

##### National Institute Of Mental Health & Neuro Sciences, Bengaluru, Karnataka, India

###### Correspondence: K. Sugavanaselvi (selvarshsugi@rediffmail.com)

*Addiction Science & Clinical Practice* 2022,**17(Suppl 1)**: O7

**Background: **Psychoactive substance use is a major cause of various illnesses affecting almost every system in the body. However,nurses in general medical care are under-utilized with regard to the detection and provision of substance use cessation interventions for patients during their routine care. The purpose of this study was to conduct a survey on the nurses' knowledge, skill, and confidence level, with regard to providing integrated interventions for psychoactive substance use, as part of the routine nursing care.

**Materials and methods: **A cross-sectional survey was conducted among nurses (N = 207) in Bruhat Bengaluru Mahanagara Palike (BBMP) hospitals from different clinical specialties. Pre designed Self-Administered Questionnaires were filled by the respondents to assess their knowledge, skill, and confidence level in delivering interventions for medical-surgical (including obstetrics) patients with substance use.

**Results: **Participants' mean age was 34.20 (SD-9.12), 71% were female. Majority (85.5%) of the nurses had not assessed their patients for addictive substance use. Only one-third had advised some patients to stop use of substances. Although some of the nurses said that they are aware of the role of addictive substances in causing various illnesses, majority (> 80%) said that they are not sure how to assist them in quitting, as part of their routine nursing care. 

**Conclusion: **The findings were used to develop a comprehensive integrated nursing intervention, which is currently being pilot-tested among the same group of nurses, and will subsequently be used to train them to provide integrated interventions in general medical settings for patients whose medical symptoms may be caused/maintained by psychoactive substance use. Keywords: Nurses, psychoactive substance use, knowledge, attitude, skill, confidence level, integrated nursing intervention, general medical settings.

### O8 Barriers for treatment and coping with alcohol use disorders (AUD)—A consumer's perspective

#### Anja Bischof*, Hannah Schmidt, Miriam Brandes, Hans-Jürgen Rumpf, Gallus Bischof

##### Department of Psychiatry and Psychotherapy, University of Lübeck, Lübeck

###### Correspondence: Anja Bischof (anja.bischof@uksh.de)

*Addiction Science & Clinical Practice* 2022,**17(Suppl 1)**: O8

**Background: **Despite a well-developed and effective treatment system, individuals with AUD are significantly less likely to seek formal help compared with people with other mental health conditions. Most studies on barriers for treatment are based on standardized surveys, which are deficient at capturing underlying causes. Reasons for non-utilization of help in qualitative studies are insufficiently known so far.

**Methods: **In the project "Alcohol-related treatment—a consumer's perspective" (ART-COPE), barriers to treatment in a biographical context were assessed with semi-structured narrative interviews. The sample consisted of 27 predominantly untreated individuals with AUD recruited in general health practices and other health settings. Interviews were transcribed and coded with MAXQDA and analyzed using qualitative content analysis. In addition, participants filled out a standardized questionnaire on barriers to treatment used in population-based surveys.

**Results: **As barriers to treatment, fear of stigmatization, shame, milieu-related factors, institutional and personal conditions, inadequate availability of treatment, negative experiences of others within treatment, a lack of drive and self-abandonment were identified. There were clear gender differences. Perceived barriers were related to problem recognition. If alcohol use was not perceived as deviating from social norms, participants did not consider seeking help and thus did not report cognizant barriers. Individually significant barriers were only insufficiently covered with the standardized questionnaire.

**Conclusions: **The central results indicate that significant factors of disease processing are not present in the sense of cognitively represented schemata and, accordingly, could not be adequately represented in previous questionnaire-based studies. Previous intervention approaches mainly focused exclusively on problem-centered, individual-focused strategies to increase the use of treatment. However, our results indicate that these approaches insufficiently correspond to the life circumstances of those affected. Instead, a careful contextualization of alcohol-related problems in various health care settings and measuring structural prevention appear to be necessary.

### O9 Brief motivational intervention combined with serious game to improve treatment retention in patients with alcohol related liver disease

#### Elsa Caballeria^1^, Clara Oliveras^1^, Maria Teresa Pons^2^, Pol Bruguera^1^, Ana Isabel López-Lazcano^1^, Soraya Sabater^1^, Cristal Martínez^2^, Laura Nuño^1^, Neus Freixa^1^, Mercedes Balcells-Oliveró^1^, Martina Pérez^3^, Anna Lligoña^1^, Antoni Gual^1^, Hugo López-Pelayo^1^

##### ^1^Grup Recerca Addicions Clínic (GRAC-GRE), Addictions Unit, Department of Psychiatry and Psychology, Clinical Institute of Neuroscience, Hospital Clínic i Universitari de Barcelona, Universitat de Barcelona, IDIBAPS, RTA (RETICS), Barcelona, Spain; ^2^Department of Psychiatry and Psychology, Clinical Institute of Neuroscience, Hospital Clínic i Universitari de Barcelona, Barcelona, Spain; ^3^Liver Unit, Hospital Clínic, Faculty of Medicine, University of Barcelona, Institut d'Investigacions Biomèdiques August Pi i Sunyer (IDIBAPS), Barcelona, Spain

Elsa Caballeria, Clara Oliveras and Maria Teresa Pons share first authorship

*Addiction Science & Clinical Practice* 2022,**17(Suppl 1)**: O9

**Background: **Adherence to addiction treatment in patients with alcohol-related liver disease (ARLD) is low, despite the harmful effects derived from alcohol use. Although some therapeutic approaches have shown promising results, the scarcity of studies prevents from determining their efficacy. Serious games (games with a therapeutic aim) have improved treatment adherence in several pathologies. We aim to explore the effectiveness of a brief therapeutic intervention to increase the retention and adherence to addiction treatment. This intervention will be based on the use of a serious game together with a brief face-to-face intervention conducted by professionals not specialists in the field of Addictions. The intervention will be designed using a co-creative approach. 

**Methods: **The co-creation process with liver specialists, patients, psychologists, psychiatrists, game designers and social workers will be organized to establish the basis for the serious games and brief motivational intervention. A pilot study (n = 10) aiming to assess the usability and feasibility of the intervention will be conducted. Next, and after incorporating the required changes to the intervention, an open randomised clinical trial will explore the retention and adherence to addiction treatment of the patients with ARLD who received the serious game-based brief intervention (n = 69) in comparison to the ones who were treated as usual (n = 69). 

**Results: **Addiction treatment retention and adherence, as well as clinical parameters such as the decrease in alcohol use, abstinence, quality of life, evolution of hepatic disease and motivation towards treatment, will be assessed at months one, three and six after the intervention. Retention to addiction treatment at six months follow-up will be the main variable. 

**Conclusions: **We aim to offer a flexible, innovative, and integrated intervention, adapted to patients' needs, and explore its efficacy to improve addiction treatment adherence and retention in patients with ARLD.

### O10 Brief online negative affect focused functional imagery training improves two-week intermediate drinking outcomes among hazardous student drinkers: a pilot study

#### Ruichong Shuai*, Alexandra E. Bakou, Jackie Andrade, Leanne Hides, Lee Hogarth

##### School of Psychology, University of Exeter, Exeter, United Kingdom

###### Correspondence: Ruichong Shuai (rs614@exeter.ac.uk)

*Addiction Science & Clinical Practice* 2022,**17(Suppl 1)**: O10

**Background: **Negative affect motivates problematic alcohol use, whereas training alternative adaptive strategies to cope with negative affect can reduce alcohol use. The current study tested whether personalised online functional imagery training (FIT) on how to use positive imagery of adaptive strategies in response to negative affect would improve intermediate drinking outcomes. 

**Methods: **Participants were 52 hazardous student drinkers who drink to cope with negative affect. Participants in the active intervention group (n = 24) were trained online over two weeks to respond to personalised negative drinking triggers by retrieving an adaptive strategy they might use in future to cope with negative affect, whereas participants in the control intervention group (n = 28) received standard risk information about binge drinking at university. Measures of daily drinking quantity, drinking motives, self-efficacy and use of protective behavioural strategies were obtained at baseline and two weeks follow-up.

**Results: **There were three significant interactions between group and time in a per-protocol analysis: the active intervention group showed increased self-efficacy over negative affect drinking and control over alcohol consumption and decreased social drinking motives from baseline to two-week follow-up, relative to the control intervention group.

**Conclusions: **These findings provide initial evidence that online training to respond to negative affect drinking triggers by retrieving images of adaptive strategies can improve intermediate drinking outcomes in hazardous, student, negative affect drinkers, whilst adding to growing beneficial effects of FIT based interventions in the field.

Already published in the International Journal of Behavioural Medicine: https://doi.org/10.1007/s12529-021-10019-9.

### O11 Community pharmacists and their roles, attitudes and knowledges about detection and advise of alcohol use in Catalonia

#### Joan Colom, Toni Veciana, Gemma Galofré, Berta Torres-Novellas, Jorge Palacio-Vieira, Lidia Segura

##### Catalan Agency of Public Health (GENCAT), Barcelona, Spain

*Addiction Science & Clinical Practice* 2022,**17(Suppl 1)**: O11

**Background: **Community pharmacists (CP) represent one of the closest health professionals to the general population and, due to accessibility, opening hours, territorial balance, and level of trust of the population; they could play a key role in the screening and brief interventions (SBI) of alcohol problems. The aim of this study is to analyse the attitudes, knowledge, and perspectives on alcohol SBI in a sample of community pharmacists in Catalonia (Spain).

**Methods: **An online survey was addressed to the CP registered in one of the four Official Pharmacists Associations of Catalonia (n = 8,027), followed by three email reminders. The questionnaire covered socio-demographic information, previous training and experience in alcohol and drugs programmes, perceptions of the CP on their abilities to perform SBI, role security (RS) and therapeutic commitment (TC) for the provision of alcohol SBI and their frequency of alcohol use (AUDIT-C).

**Results: **The final response reached 7.9% (n = 639), 79.9% were women, 63.9% were 41 years or older and more than the half had 21 years of experience. Twenty-five percent of respondents had received training on alcohol or drugs during the last five years and only 9.1% participated in any alcohol or drugs prevention program. Scores of TC were higher than RS (18.1 vs. 13.2) and both measures were higher among men, those who received previous training, those pharmacies with fewer workers and those who had participated in other preventive or public health initiatives. Abilities to perform SBI were higher among older CP (56 years or more) and those with higher scores of TC and RS.

**Conclusions: **This is the first study aimed at the analysis of SBI at community pharmacies in Catalonia. The design and implementation of alcohol SBI initiatives in community pharmacies need intensive training of their professionals, specially to increase their perception of role security and therapeutic commitment.

### O12 Delivering adolescent SBIRT as part of a cluster randomized trial in rural U.S. health centers: provider fidelity to delivery of the intervention

#### Shannon Gwin Mitchell, Laura B. Monico, Jan Gryczynski, Mishka Terplan

##### Friends Research Institute, Baltimore, Maryland, USA

*Addiction Science & Clinical Practice* 2022,**17(Suppl 1)**: O12

**Background: **This presentation will include an update of an on-going stepped-wedge randomized trial of adolescent SBIRT. The FaCES intervention includes a prescribed set of responses (anticipatory guidance, abbreviated BI, full BI) based on S2BI screening results. Adolescent patients, age 12–17 years, receive either the FaCES intervention or standard care, depending on when their provider is randomized and trained.

**Method**s: As of February 2021, a total of 1,239 patients had been recruited into the study and 14 of 20 providers had been trained to deliver FaCES across 5 U.S. health centers in two states—New Mexico (NM) and Tennessee (TN). Of those, 938 patients saw an untrained provider and 301 saw a provider trained to deliver FaCES. Screening results were compared with provider checklist responses to gauge fidelity of intervention delivery based on S2BI risk level.

**Results: **Among the 1,239 participants enrolled there were significant differences between states in patient distribution of female gender (57% TN vs. 47% NM, p = 0.001), white race (88% TN vs. 44% NM, p < 0.000), and Hispanic ethnicity (5% TN vs. 69% NM, p < 0.000). At baseline, past year use of tobacco, alcohol, and marijuana were reported by 19%, 20%, and 16% of adolescent patients, respectively. Fourteen providers were trained to deliver the FaCES intervention (6 physicians and 8 physician's assistants/nurse practitioners). Ninety-eight percent of patients with no reported substance use received anticipatory guidance, while 76% of those reporting past year substance use received a BI (9% of patients declined). A change plan was documented for 53%. Among patients who qualified for a BI, 77% appropriately received abbreviated BI and 49% received full BI.

**Conclusions: **Pediatric providers trained for the present study are utilizing their clinical judgement and providing patient-centered SBIRT services, which is encouraged in the FaCES intervention. Study findings will need to take such factors into consideration.

### O13 Delivering brief intervention in group: strategies to assess harmful alcohol use at primary health care

#### Erika G. L. Ramírez*, Divane de Vargas, Stephen Strobbe

##### Nursing School of São Paulo University, São Paulo, Brazil

###### Correspondence: Erika G. L. Ramírez (egleonr@usp.br)

*Addiction Science & Clinical Practice* 2022,**17(Suppl 1)**: O13

**Background: **The problematic alcohol use in the male population continues to be a public health problem in the world. In Brazil, the prevalence of harmful alcohol use in a population over 15 years old is 5.2% for women, while among men it is 20.7%. In this sense, the application of Brief Intervention (BI) has been suggested as an effective strategy in changing the risk and harmful alcohol use in the population. More recently, due to the limitations of individual IB, regarding the optimization of human resources and greater coverage in health services, the Brief Group Intervention (BGI) has been proposed as a strategy with the potential to face these limitations. The aim of this study was to evaluate the effectiveness of the BGI in reducing the alcohol in men with risk and harmful alcohol use treated in primary health care.

**Materials and methods: **Randomized clinical trial, with a follow-up of 30 and 90 days, conducted in a primary health care unit in Brazil. Were randomized 112 men, of which 55 were allocated to the experimental group and 57 to the control group. The alcohol use was assessed in both groups, using the Alcohol Use Disorders Identification Test (AUDIT). The experimental group received a brief group intervention session with the FRAMES model, and the control group was instructed to continue with the usual care of the unit. The data were analyzed using the GEE method (Generalized Equations Estimating).

**Results: **A significant difference (p < 0.001) between the experimental (GE) and control (CG) groups was evidenced, showing a decrease in alcohol use in the GE.

**Conclusions: **The results of this study suggest the application of IBG is an effective approach with the potential to reduce alcohol use in the male population at primary health care settings.

**Trial Registration: **Registered in the Brazilian Clinical Trials Registry/Registro Brasileiro de Ensaios Clínicos (ReBEC). REBEC registration number: RBR-6zk87m-UTN number: U1111-1205-8313.

### O14 Development of a training module on substance abuse awareness and prevention for field functionaries working in grassroots communities

#### Sonia Sain*, Aparna Khanna

##### Department of Development Communication and Extension, Lady Irwin College, University of Delhi, New Delhi, India

###### Correspondence: Sonia Sain (soniasain174@gmail.com)

*Addiction Science & Clinical Practice* 2022,**17(Suppl 1)**: O14

**Background: **Substance abuse is a sensitive issue and lot is to be done to improve the knowledge, communication and counselling skills of field functionaries because they are the frontline workers and key persons who are directly involved with the communities. The study aims to assess the training needs of field functionaries working in grassroots communities on the issue of substance abuse. The study will find what knowledge and skills are required by the field functionaries and challenges they face while doing their job. An assessment of their training needs would help in designing of a training module on the issue of substance abuse. 

**Methods: **The population of the study consisted all functional organisations working directly with adolescents and youth (13–35 years of age) on any development programme or issue. Purposive sampling technique was used to select the sample in Delhi and NCR. Total of 120 field functionaries, 30 NGO Heads and Management Staff and 10 experts from the field of substance abuse were included in the study. Semi-structured interview schedules has been prepared for different set of respondents. The data obtained from different groups were compiled and analysed in various aspects of the study. 

**Results and conclusion: **The major findings of the study shows that there is no pre-service and in-service training given to field functionaries on the issue of substance abuse and it causes difficulty in working in the community because field functionaries has not sufficient knowledge related to substance abuse. Drug addiction is too much in the communities and people need information on how to prevent and overcome it. Field functionaries expressed a need for training and information should be given through innovative methods. Content suggested for training programme can be basic knowledge about drugs and their physical and psychosocial effects, preventive measures, Rehabilitation centers and their address.

### O15 Development of an informed text-message library and telephone health coaching intervention for diverse community health center patients who use drugs in Los Angeles

#### Dallas Swendeman^1,*^, Stephanie Sumstine^1^, Zachary Jacobs^2^, Efren Aguilar^2^, Whitney Akabike^2^, Lillian Gelberg^2^

##### ^1^Department of Psychiatry and Biobehavioral Sciences, David Geffen School of Medicine, UCLA, Los Angeles, USA; ^2^Department of Family Medicine, UCLA, Los Angeles, USA

###### Correspondence: Dallas Swendema (dswendeman@mednet.ucla.edu)

*Addiction Science & Clinical Practice* 2022,**17(Suppl 1)**: O15

**Background: **The objective of this analysis was to identify barriers and facilitators to drug use reduction to inform development of content for theory-based automated feedback text-messages, sent in response to weekly self-monitoring surveys, and to inform telephone health coaching for patients with moderate risk drug use (ASSIST score 4–26; ASAM 0.5) in the new NIDA-funded QUIT-Mobile study.

**Methods: **Two researchers conducted thematic content analysis of the health educator coaching log data from the original Quit Using Drugs Intervention Trial (QUIT) and the subsequent Binational QUIT (LA and Mexico). Common themes were identified through iterative rounds of coding and discussion with the study team. 

**Results: **The most commonly cited barriers to reducing or stopping drug use were relaxation and increased QoL; works better than prescribed medication; pain relief; sleep; peers/social environment. Feedback messages were developed for the most commonly cited facilitators for drug use reduction, which overlapped with barriers, and all codes were collapsed into general "domains" patients would have the opportunity to opt-in to (ex. Health, Activities, Social, Lifestyle, Positive Future Orientation, and Services). Each code was also assigned as a "Motivator" or "Technique/Strategy" to support patients in maintaining their drug use reduction goals. Messages were developed based on Social Cognitive Theory, Health Belief Model, and Social Support Theory and aimed to provide tailored feedback messages with affirmations, motivational text messages, and drug use reduction technique tips. Messages were also drawn and adapted from text-message libraries used in prior studies to reduce substance use in diverse communities.

**Conclusion: **Results from qualitative data analysis suggest there are unique barriers and facilitators to drug use reduction in diverse low-income primary care patients. Feedback messages in interventions utilizing mobile self-monitoring should be tailored to individuals' noted barriers and facilitators to enhance motivation and reinforce alternatives to drug use.

### O16 Development and feasibility testing of an intervention to help women in the community to quit addictive substance use

#### T.S. Sunitha, Prasanthi Nattala, Girish N. Rao, K. S. Meena 

##### National Institute of Mental Health and Neuro Sciences, Bengaluru, Karnataka, India

*Addiction Science & Clinical Practice* 2022,**17(Suppl 1)**: O16

**Background: **The use of alcohol and smokeless tobacco among women in India is rising, however, there are no known structured interventions and testing their feasibility. The purpose of this study was to develop an intervention for women to help them quit addictive substance use (ASU), based on their own narratives as to what help they need to quit.

**Methods: **An intervention was developed for women from selected rural and urban communities in Bangalore, Karnataka. The intervention comprises of: a) Information on the adverse health impact of ASU (including during pregnancy and lactation), specifically smokeless tobacco and alcohol, through graphic illustrations (b) PowerPoint video to clarify myths and misconceptions (c) Ways of quitting ASU, presented through a video (d) Adaptive coping, demonstrated through a short film.

**Results: **Feasibility testing of the Intervention is currently ongoing in the same communities.

**Conclusion: **The study findings can help to understand the relevance and acceptability of the developed Intervention among the women. Based on this, the Intervention can be refined and finalized for women in the community, to help them quit ASU.

### O17 Development, feasibility, and preliminary validation of a Spanish language version of the TAPS Tool for substance use screening in primary care

#### Jan Gryczynski^1^, Katherine Sanchez^2^, Steven B Carswell^1^, Robert P Schwartz^1^

##### ^1^Friends Research Institute, Baltimore, USA; ^2^School of Social Work, University of Texas at Arlington, Arlington, USA

*Addiction Science & Clinical Practice* 2022,**17(Suppl 1)**: O17

**Background: **The TAPS Tool, developed through the U.S. National Institute on Drug Abuse Clinical Trials Network, is a validated two-stage screening and brief assessment for unhealthy substance use in primary care. Although the U.S. has a large Spanish-speaking population, the TAPS has only been validated in English.

**Methods: **We developed a Spanish language version of the TAPS Tool and conducted a small study of its feasibility, acceptability, and preliminary validity. Participants were adult primary care patients ages 18 or older with Spanish as their primary language (N = 10 for development/refinement using qualitative cognitive interviewing; N = 100 for the preliminary validation study). The TAPS Tool was examined in interviewer-administered and electronic self-administered formats, with participants assigned at random to the order of administration. We examined disclosure of substance use on the TAPS by administration format, and compared it with established measures (modified CIDI; WHO ASSIST).

**Results: **The Spanish language TAPS was feasible to use and participants reported high levels of acceptability. The sample had low rates of past 12-month substance use (11% tobacco, 28% risky alcohol, 4% illicit drugs, 1% non-medical prescription drugs) and use disorders (7% tobacco, 2% alcohol, 1% other substances). The self-administered TAPS elicited 1, 3, and 1 additional disclosures of tobacco, risky, alcohol, and marijuana use than the interviewer-administered TAPS, respectively. Rates of disclosure on the TAPS were similar to those on established measures for past 12-month and 3-month time frames.

**Conclusions: **The current study represents a starting point for expanding the availability of the TAPS Tool beyond its original English language version into Spanish. The Spanish language TAPS Tool could expand options for substance use screening in primary care settings with Spanish-dominant/preferred populations. Future directions for validating and disseminating the Spanish-language TAPS will be discussed.

### O18 Drinking risk situations and strategies to deal with them reported by Brazilian users of the Bebermenos" (Drinkless) e-health program

#### Maria L. O. Souza-Formigoni^1^*, Micaella L. Silva^2^, Keith M. Soares^3^

##### ^1^Universidae Federal de Sao Paulo, Brazil; ^2^Graduate Program on Psychobiology, Universidae Federal de Sao Paulo, Brazil; ^3^Associação Fundo de Incentivo a Pesquisa (AFIP), Sao Paulo, Brazil

###### Correspondence: Maria L. O. Souza-Formigoni (mlosformigoni@unifesp.br)

*Addiction Science & Clinical Practice* 2022,**17(Suppl 1)**: O18

**Background: **In a study supported by the WHO Department of Mental Health and Substance Abuse, in partnership with researchers from the Netherlands, Belarus, India and Mexico, we developed an e-health screening and intervention directed to alcohol risk users, named in Portuguese "Bebermenos" (Drinkless).

**Methods: **Brazilian alcohol users, classified according to their AUDIT (Alcohol Use Disorders Inventory Test) scores as "at-risk" or "suggestive of dependence" were included in the program. Among the diverse tools available, one was the record. Of their most common drinking situations followed by intended strategies to avoid drinking, or at least to do it in a low risk pattern. Using a qualitative approach (content analysis), in the present study we categorize the main drinking situations and strategies to deal with them reported by the users of the program. We classified the drinking situations according to the 8 categories proposed by Annis et al. (1982). The classification of strategies was defined by consensus among three researchers who had previously classified them, independently. We analyzed data from 130 participants who had filled out those forms.

**Results: **The most prevalent categories of drinking situations were: Pleasant Times With Others, Social Pressure to Drink, Unpleasant Emotions and Urges and Temptations. As regards the strategies to avoid drinking, the most frequently reported were: increase water or soda ingestion, reject events in which there are alcoholic beverages, share the decision to stop drinking with others, practice physical activity, hang out with people who drink under control or do not drink, and stay at home. At follow-up they reported this kind of "exercise" was helpful to control their drinking.

**Conclusion: **These data indicate the importance of encouraging alcohol problem users to be aware of their drinking risk situations and to thinking about them in advance, in order to develop better strategies to deal with them and prevent relapses.

**Trial Registration: **This study was developed with data from the Brazilian participants of the project “Alcohol e-Help self-help intervention”. Trial registration: ISRCTN14037475, https://doi.org/10.1186/ISRCTN14037475

### O19 Effect of health behaviours on risky drinking amongst adolescents in the UK: Secondary data analysis from the SIPS JR-HIGH pilot study in North East England

#### Srinidhi Koya, Dorothy Newbury- Birch

##### School of Social Sciences, Humanities and law, Teesside University, Middlesbrough, UK

*Addiction Science & Clinical Practice* 2022,**17(Suppl 1)**: O19

**Background: **Risky drinking continues to be the leading cause of death and disability-adjusted life-years amongst young people, globally. The study examined the effect of health behaviours on risky drinking amongst adolescents aged 14–15 years old in North East England, UK.

**Methods: **Secondary data analysis of the NIHR PHR-funded SIPS JR-HIGH pilot study was conducted using the baseline survey data. The questionnaire consisted of tools used for alcohol screening (A-SAQ), measuring risky alcohol use (AUDIT), a general lifestyle questionnaire (diet, smoking, sexual behaviour, and exercise), and general psychological health (WEMWBS). Descriptive and analytical statistics were obtained using measures of central tendency, proportions, and logistic regression.

**Results: **Amongst the 1280 respondents who took part in the study, half were females and the majority belonged to the White ethnic group. While half of the respondents reported adequate fruit and vegetable (F&V) consumption, only 10% had adequate levels of physical activity and one-third of them were smokers. A quarter of the respondents had AUDIT-C scores ≥ 5 and 40% of the respondents had drunk more than 3 units of alcohol in the past 6 months. Logistic regression showed that females (OR: 1.49, p-value: 0.001) and adolescents with inadequate F&V consumption (OR: 1.32, p-value: 0.015) had higher odds of risky drinking. Non-smokers had lower odds of risky drinking (OR: 0.124, p-value: < 0.001). Also, females had higher odds of having an inadequate physical activity (OR: 1.76, p-value: 0.005), lower odds of abstaining from smoking (OR: 0.51, p-value: < 0.001) and, lower odds of mental well-being (OR: 0.51, p-value: < 0.001), than males. 

**Conclusion: **To develop successful interventions for risky drinking amongst adolescents, future studies need to address these determinants. My PhD study will examine the interaction between these determinants and alcohol consumption as a result of socio-cultural factors.

### O20 Effects of adolescent SBIRT education using simulated learning technology in health professional training

#### Tracy McPherson*, Hildie Cohen, Weiwei Liu, Jackie Sheridan

##### Public Health Department, NORC at the University of Chicago, Chicago, USA

###### Correspondence: Tracy McPherson (Mcpherson-tracy@norc.org)

*Addiction Science & Clinical Practice* 2022,**17(Suppl 1)**: O20

**Background: **Substance use in adolescence is linked to a range of negative life consequences. Consequently, there is a great need for social workers, nurses, and other health professionals to help prevent and reduce substance use with youth. NORC at the University of Chicago along with leading professional education associations and experts partnered to develop and test an adolescent screening, brief intervention, and referral to treatment (SBIRT) curriculum for use in nursing, social work, and interprofessional education. Since 2015, the curriculum has been implemented with more than 10,000 students in over 200 programs in the U.S. This study evaluated the impact of the education on students' attitudes towards working with people who drink alcohol; perceived readiness, confidence, and competence; knowledge and skills.

**Materials and methods: **Students completed a pre-training survey, received adolescent SBIRT education including an online simulation training, and a post-training survey. A pretest–posttest within-subjects design was used to investigate the effects on student attitudes; confidence, competence, and readiness; knowledge and skills. Differences between groups were also explored for program-level variables using OLS Regression. The sample included 18 schools with 556 students. The analysis compared subgroups (e.g., undergraduate/graduate, prior training) on pre-post differences in outcomes using independent sample t-tests and OLS regressions. Through review of implementation progress reports, qualitative data was analyzed to assess the impact of implementation format and dosage on training outcomes. 

**Results: **Outcomes on perceived confidence, competence, readiness, knowledge and skills were significantly higher after SBIRT education. Implementation format and dosage did not significantly impact outcome measures when controlling for other participant demographic covariates. 

**Conclusions: **The findings suggest that adolescent SBIRT education including simulation-based training can positively affect student outcomes as they prepare to implement adolescent SBIRT in the field. The findings can also inform educators on the differences in outcomes among groups and inform curriculum infusion.

### O21 Effectiveness, cost-effectiveness and cost-utility of a digital smoking cessation intervention for cancer survivors: health economic evaluation and outcomes of a pragmatic randomized controlled trial

#### Ajla Mujcic^1,2^, Matthijs Blankers^1,3,4^, Brigitte Boon^5,6^, Irma M. Verdonck-de Leeuw^7,8^, Filip Smit^1,8,9^, Margriet van Laar^1^, Rutger Engels^2^

##### ^1^Trimbos Institute, The Netherlands National Institute of Mental Health and Addiction, Utrecht, The Netherlands; ^2^Erasmus School of Social and Behavioural Sciences, Erasmus University Rotterdam, Rotterdam, The Netherlands; ^3^Department of Research, Arkin Mental Health Care, Amsterdam, The Netherlands; ^4^Department of Psychiatry, Amsterdam UMC, Location AMC, University of Amsterdam, The Netherlands; ^5^Department Scientific Research, Academy het Dorp, Arnhem, The Netherlands; ^6^Department Scientific Research, Siza, Arnhem, The Netherlands; ^7^Department of Otolaryngology-Head and Neck Surgery, Amsterdam UMC, Cancer Center Amsterdam, Amsterdam, The Netherlands; ^8^Department of Clinical, Neuro and Developmental Psychology, Amsterdam Public Health research institute, Vrije Universiteit, Amsterdam, The Netherlands; ^9^Department of Epidemiology and Biostatistics, Amsterdam Public Health research institute, Amsterdam UMC, location VUmc, Amsterdam, The Netherlands

*Addiction Science & Clinical Practice* 2022,**17(Suppl 1)**: O21

**Background: **Smoking cessation (SC) interventions may contribute to the wellbeing of cancer survivors.

**Methods: **A health economic evaluation alongside a pragmatic two-arm parallel-group randomised controlled trial (RCT) with follow-ups at 3, 6 and 12 months comparing a digital interactive SC intervention compared to a non-interactive online information brochure for cancer survivors. The study was conducted from November 2016 to September 2019. Participants were Dutch adult cancer survivors who were current smokers with the intention to quit smoking. In total, 165 participants were included and analysed; 83 in the intervention group and 82 in the control group. Primary outcome was self-reported 7-day smoking abstinence at 6-month follow-up. Secondary outcomes were quality adjusted life years (QALYs) gained, number of cigarettes smoked, nicotine dependence, and treatment satisfaction. For the health economic evaluation, healthcare costs, and societal costs (including productivity losses and intervention costs) and effects were assessed over a 12-month horizon.

**Results: **At 6-month follow-up the quit rates were 27.7% (n = 23) and 25.6% (n = 21) in the MyCourse and control group, respectively (OR = 0.47 95%CI 0.03 to 7.86, P = 0.60). The number of cigarettes decreased more over time and the MyCourse group demonstrated a significantly greater reduction at 12-month follow-up (IRR = 0.87, 95% CI 0.76 to 1.00, P = 0.04). In the cost-utility analysis, the MyCourse intervention was not preferred over the control group when taking the societal perspective. In addition, with actual smoking behaviour as the outcome, the MyCourse group led to marginally better results per reduced pack-year against higher societal costs, with a mean incremental cost-effectiveness ratio of US$52,067 (95% CI 32,515 to 81,346).

**Conclusions: **At 6 months, there was no evidence found for a differential effect on cessation rates. At 12 months, the MyCourse intervention led to a greater reduction of number of smoked cigarettes at higher costs compared to the control group.

### O22 Effectiveness of a nurse-conducted brief interventions in patients with alcohol use disorders admitted in gastroenterology ward, using a house-to-house survey—A case–control study

#### Johnson R. Pradeep^1^, Abiya N. Vinnarasi^1^, Harshad Devarbhavi^2^, Sumithra Selvam^3^, Ashok Mysore^1^*

##### ^1^Department of Psychiatry, St. John's Medical College, Bengaluru, Karnataka, India; ^2^Department of Gastroenterology, St. John's Medical College, Bengaluru, Karnataka, India; ^3^Division of Epidemiology, Biostatistics & Population Health, St. John's Research Institute, St. John's Medical College, Bengaluru, Karnataka, India

###### Correspondence: Ashok Mysore (ashok.mv@stjohns.in)

*Addiction Science & Clinical Practice* 2022,**17(Suppl 1)**: O22

**Background: **In India, 14.6% of the population use alcohol, among them 5.2% are problem drinkers and it is a huge burden. Alcohol use disorders (AUD) have the widest treatment gap and one important reason is the shortage of specialists in India. Hence, task-shifting to health care professionals such as Nurses to deliver brief interventions is needed. There are limited studies where a house-to-house follow-up has been carried out to assess the drinking status in India. This study aimed to assess the effectiveness of Nurse-Conducted Brief Intervention (NCBI) using a house-to-house survey to objectively confirm the drinking status of the patients.

**Methods: **Using a case–control design, 51 patients from the Intervention Group (IG) and 20 in the Non-Intervention Group (NIG) were selected from the BIG service. A House-to-house survey was carried out by a trained Research Assistant (RA). The RA administered a semi-structured proforma to capture sociodemographic data and alcohol use-related data such as drinking status, PHQ-9, and AUDIT questionnaire. 

**Results: **Significant reduction in the median AUDIT score was found in the IG during the visit (Before intervention = 15 [IQR = 8–21) vs. House visit = 12 [IQR 9–14], p value = 0.005), compared to NIG (Before intervention = 10 [IQR 9.25–11] vs. House visit 9.50 [IQR 9–11], p value = 0.371). There were significantly more number of abstainers in the IG (NIG n = 14 vs. IG = 48) and few relapsers (NIG n = 6 vs. IG n = 3) compared to NIG (p = 0.012). The odds ratio of those who abstained due to the NCBI was 2.33.

**Conclusions: **The findings from this study support the effectiveness of an NCBI in patients with AUD using an objective method (House-to-house) of confirmation of the drinking status.

### O23 Efficacy of Brief Intervention among university students with different levels of severity of alcohol use related problems

#### Paula V. Gimenez, Mariana Cremonte, Raquel Peltzer, Tomás Salomón, Karina Conde

##### Research Group on Psychoactive substances and injuries-Institute of Basic, Applied Psychology and Technology (IPSIBAT), National Scientific and Technical Research Council (CONICET), National University of Mar del Plata (UNMdP), Mar del Plata, Argentina

*Addiction Science & Clinical Practice* 2022,**17(Suppl 1)**: O23

**Background: **Brief Intervention (BI) is often considered less effective among people with higher alcohol use related problems, but the evidence is mixed. We evaluated the efficacy of BI with and without Normative Feedback (NF) in reducing alcohol consumption among university students with different severity levels.

**Methods: **821 students from a national public university in Argentina were randomized into BI without NF (BI), BI with NF (BI-NF) or a screening control group (CG). Students with heavy episodic drinking (HED) in the last 12 months were included, while those with dependency indicators were excluded. Three months later they were re-assessed (n = 537). The outcomes were: number of standard units (SU) consumed per occasion, drinking frequency, and HED frequency. Analyses (negative binomial Poisson and ordinal regressions) were performed in subgroups according to severity (high: AUDIT scores 7–14; low: AUDIT scores 1–6). 

**Results: **Among those with high severity, BI-RN reduced significantly quantity per occasion, (Waldχ2 = 8.51, OR = 0.74, CI 95% 0.6 to 0.9, p = 0.004) compared with CG. BI compared with CG, reduced the frequency of consumption (Waldχ2 = 6.9, OR = 0.34, CI 95% 0.15 to 0.76, p = 0.009). There were no significant differences in any outcome between BI and BI-RN. Among those with low severity, BI-RN compared to CG was effective to reduce quantity per occasion (Waldχ2 = 13.11, OR = 0.7, CI 95% 0.57–0.85, p = 0.001), drinking frequency (Waldχ2 = 13.99, OR = 0.3, CI 95% 0.16–0.57, p = 0.001) and HED frequency (Wald χ2 = 16.41, OR = 0.19, CI 95% 0.08–0.42, p = 0.001). BI compared to CG also reduced quantity per occasion (Waldχ2 = 21.98, OR = 0.7, CI 95% 0.6–0.81, p = 0.001), drinking frequency (Waldχ2 = 14.67, OR = 0.34, CI 95% 0.19–0.59, p = 0.001) and HED frequency (Waldχ2 = 54, OR = 0.06, CI 95% 0.03–0.12, p = 0.001). When comparing BI with BI-RN, there was a significant difference in HED frequency favoring BI participants (Waldχ2 = 7.53, OR = 0.3, CI 95% 0.13–0.71, p = 0.006). 

**Conclusions: **High severity students benefit more from a BI-RN. However, BI is more appropriate for less severe, since this is a shorter intervention with similar results.

### O24 Efficacy of a novel brief motivational intervention for alcohol-intoxicated young adults in the emergency department: a randomized control trial

#### Jacques Gaume^1^, Nicolas Bertholet^1^, Molly Magill^2^, Jim McCambridge^3^, O. Hugli^1^, Jean-Bernard Daeppen^1^

##### ^1^Lausanne University Hospital, Lausanne, Switzerland; ^2^Brown University, Providence, Rhode Island, USA; 3 University of York, York, England

*Addiction Science & Clinical Practice* 2022,**17(Suppl 1)**: O24

**Background: **Heavy drinking among young adults is a major public health concern. Brief motivational interventions (bMI) in the Emergency Department (ED) have shown promising but inconsistent results.

**Methods: **We developed a novel bMI model with in-person discussion in the ED plus up to three booster telephone calls and tested its efficacy in a randomized controlled trial. Young adults (18–35 years old) admitted to the ED with alcohol intoxication (N = 344) were randomized to receive either bMI or a minimal intervention (structured brief advice in the ED). Follow-up questionnaires were conducted at 1-, 3-, 6-, and 12-months. Primary outcomes were the number of heavy drinking days (HDD, i.e. 6 standard drinks or more) over the previous month and the total score of the Short Inventory of Problems (SIP) questionnaire over the previous 3 months. Secondary outcomes included weekly drinking amount, additional alcohol consequences, change in Alcohol Use Disorder Identification Test (AUDIT) score, readmission to the ED, and alcohol treatment initiation. 

**Results: **Using generalized estimating equations, we observed an overall increase of HDD over the follow-up time (B = 0.04, 95% CI 0.02–0.05, p < 0.001) and a significant time X intervention interaction (B = − 0.03, 95% CI − 0.05–0.003, p = 0.03), indicating that the bMI group showed statistically less increase in HDD compared to the brief advice group. Differences were non-significant for SIP score and secondary outcomes except for alcohol treatment initiation which was significantly more likely in the bMI group over the 12-month follow-up (OR = 3.70, 95% CI 1.07–12.78, p = 0.04). 

**Conclusions: **This study showed that a bMI model implemented in an ED context might help young adults admitted with alcohol intoxication to maintain a lower level of HDD over one year. Our intervention also increased the likelihood of initiating specialized alcohol treatment. Results were inconclusive regarding alcohol problems and consequences, weekly drinking amount, AUDIT score, and readmission to the ED.

### O25 Exploration of college students' perceptions regarding cannabis use: a community study from Bangalore, south India

#### Kannan K^1^, Prasanthi Nattalla^1,2^*, Jayant Mahadevan^2 3^, KS Meena^4^

##### ^1^Department of Nursing, National Institute of Mental Health and Neuro Sciences, Bengaluru, Karnataka, India; ^2^Center for Addiction Medicine, National Institute of Mental Health and Neuro Sciences (NIMHANS), Bengaluru, India; ^3^Department of Psychiatry, National Institute of Mental Health and Neuro Sciences (NIMHANS), Bengaluru, India; ^4^Department of Mental Health Education, National Institute of Mental Health and Neuro Sciences (NIMHANS), Bengaluru, India

Corresondence: paidi89@gmail.com

*Addiction Science & Clinical Practice* 2022,**17(Suppl 1)**: O25

**Background: **Cannabis use (e.g. bhang, ganja, and charas) among college students is increasing in India, yet research regarding their viewpoints on how they can be helped is scarce. The aim of this study is to explore the perceptions of college students regarding the use of cannabis among students, and the kind of help they need in this regard.

**Method: **Focus group discussions were conducted with college students (both government and private) (N = 43), to identify their viewpoints on how the students can be helped to quit cannabis use. The audio-recorded discussions were transcribed verbatim, themes and sub-themes identified.

**Results: **Four major themes (with subthemes) were identified: (1) Patterns of cannabis use among college students (commonly used substances [including street names], age of initiation, methods of using, reasons for using), (2) Perceptions about potential impact of cannabis use on students—health, relationships, academics, social and interpersonal (3) Perceptions regarding medical and legalization of cannabis use (4) Measures to prevent/reduce cannabis use among college students.

**Conclusion: **The information from this study can guide the development of an intervention that is relevant to the specific needs of the college students. This can be important, as interventions are likely to be maximally effective when they are informed by the end-users themselves, viz. college students.

### O26 Factor structure and diagnostic properties of a digital version of the AUDIT compared to DSM-V and Timeline Follow Back in current drinkers in Chilean primary care

#### Nicolas B. Lantadilla^1^, Pablo N. Cárdenas^2^, Fernando P. Arrué^1^

##### ^1^Pontificia Universidad Católica de Chile, Santiago, Chile; ^2^Ministerio de Salud Chile, Santiago, Chile

*Addiction Science & Clinical Practice* 2022,**17(Suppl 1)**: O26

**Background: **We studied the factor structure and diagnostic accuracy of a digital version of the Alcohol Use Disorders Identification Test (d-AUDIT) in Spanish.

**Methods: **Primary care users who consumed alcohol six or more times in the last year (n = 471) first completed the d-AUDIT on 7-inch tablets and then were interviewed to complete a 1-year Timeline Followback and the alcohol module from the SCID—DSM-V. Confirmatory factor analyses explored the underlying structure, and areas under the ROC curve (AUC) analyzed the accuracy of various scores to detect binge-drinking and alcohol use disorder (AUD).

**Results: **Participants completed the d-AUDIT in 2.9 min (SD 1.3). The two- and three-factor solutions presented satisfactory overall fit, with RMSEA of 0.04 (0.01–0.05) and CFI and TLI 0.95. All the estimators were statistically significant, and the indicator loads were in the middle-high range, leading to interpretable factor structures. The correlations among the consumption factor and the consequence and dependency factors were low in both solutions (between 0.35 and 0.38). The total score presented an AUC of 0.94 (CI 0.91, 0.97) for severe AUD, outperforming the AUDIT-C (consumption items, p < 0.001) for any AUD but performing similarly to the FAST (binge + role + black-outs + concern items). All measures showed similar AUCs for binge-drinking (AUDIT 0.82 [CI 0.76,0.88], AUDIT-C 0.82 [CI 0.76,0.87], and FAST 0.78 [CI 0.72, 0.84]). At a threshold of 7, sensitivity and specificity for AUD were 0.84 and 0.73, and at a threshold of 4, sensitivity and specificity were 0.85 and 0.53 for binge-drinking.

**Conclusions: **A two-factor solution is more parsimonious and captures two empirical dimensions of alcohol use in primary care: harmful consumption and consequences/dependency. The FAST, a short version that combines these two factors, performed better than the AUDIT-C.

### O27 False negatives in urine drug screening to inform directed expansion of screening, brief intervention, and referral to treatment (SBIRT) in trauma patients

#### Stephanie T. Weiss*, Jeffrey A. Brent, William McGill, Laura J. Veach

##### Wake Forest School of Medicine, North Carolina, USA

###### Correspondence E-mail: Stephanie T. Weiss (stweiss2@gmail.com)

*Addiction Science & Clinical Practice* 2022,**17(Suppl 1)**: O27

**Background: **To reduce trauma recidivism, United States trauma centers are required to screen hospitalized trauma patients for problematic alcohol use, intervene, and refer to treatment. However, no similar requirement exists for other substance use besides alcohol. Relying on urine drug screen (UDS) results to screen for substance use underestimates the rate of problematic substance use since many substances are not detectable by UDS. The objective of this study was to estimate the types and rates of false negative (FN) results in trauma patient UDS compared with confirmatory testing by liquid chromatography-mass spectrometry (LC–MS). 

**Methods: **We performed a prospective cohort pilot study of deidentified urine samples from adult trauma and burn activation patients. Samples were analyzed by a professional reference laboratory that provides LC–MS confirmatory testing of > 200 analytes along with an immunoassay-based UDS. Patients who did not provide urine samples while in the Emergency Department were excluded. Iatrogenic medications given by the treating team were documented and excluded from the total count of FN as these substances could not have contributed to the patient's traumatic event.

**Results: **100 urine samples meeting inclusion criteria were analyzed. Psychoactive FNs were detected in 56/100 samples, with the most frequent non-iatrogenic psychoactive substance classes including anticonvulsants (primarily gabapentin, N = 13), pharmaceutical opioid agonists (N = 12), antihistamines (primarily diphenhydramine, N = 11), and phenethylamines (primarily bupropion, N = 5). Non psychoactive FNs were detected in 70/100 samples, with the most common nonpsychoactive FNs being nicotine (N = 33), caffeine (N = 23), acetaminophen (N = 22), and antidepressants (N = 12).

**Conclusions: **Analogous to the current efforts to provide SBIRT for trauma patients who misuse alcohol, our results provide evidence in favor of extending SBIRT to trauma and burn activation patients with problematic use of other substances. Expansion of SBIRT efforts appears to be particularly needed for misuse of tobacco products, prescription analgesics, and over-the-counter antihistamines in this patient population.

### O28 Outcome reporting in brief intervention trials: Alcohol' (ORBITAL) core outcome set: from international consensus to international use?

#### Dr. Gillian Shorter, Jeremy Bray, Nick Heather, Anne H Berman, Emma L Giles, Mike Clarke, Carolina Barbosa, Amy O'Donnell, Aisha Holloway, Heleen Riper, Jean-Bernard Daeppen, Maristela G Monteiro, Richard Saitz, Jennifer McNeely, Lela McKnight-Eily, Alex Cowell, Paul Toner, Dorothy Newbury-Birch.

##### Queen's University Belfast, Northern Ireland

*Addiction Science & Clinical Practice* 2022,**17(Suppl 1)**: O28

**Background: **INEBRIA members developed the 'Outcome Reporting in Brief Intervention Trials: Alcohol' (ORBITAL) core outcome set (COS) for efficacy and effectiveness of trials and evaluations for alcohol brief interventions (ABIs). Here we summarise this process and discuss how we might improve uptake and use in the INEBRIA and ABI community.

**Methods: **We used the COMET Initiative methodology. This involved a systematic review which identified 2641 outcomes in 401 ABI papers measured by 1560 different approaches. These outcomes were aggregated into outcome categories, and 150 participants from 19 countries participated in a two-round e-Delphi outcome prioritization exercise which identified 15 of 93 outcome categories to be discussed at a consensus meeting of key stakeholders. A psychometric evaluation determined how to measure the core outcomes and we draw on established COS for dissemination strategies.

**Results: **Ten outcomes with measures form the ORBITAL COS: typical quantity, typical frequency, frequency of heavy episodic drinking, a combined consumption measure summarizing alcohol use, hazardous or harmful drinking (average consumption), standard drinks consumed in the past week (current consumption), alcohol-related consequences, alcohol-related injury, use of emergency healthcare services (impact of alcohol use), and quality of life. To support potential users, we created a data dictionary, used open science for transparency and to support implementation, and have plans to disseminate widely to individuals and groups.

**Conclusion: **The ORBITAL COS is a consensus standard for future trials/evaluations of ABIs as the recommended minimum and does not limit other outcomes relevant to your trial. It can improve synthesis of new trial findings, between-study comparisons, citations/use of research, and enhance utility of findings for decision makers. It can reduce redundant or selective measurement (reporting only some, usually significant outcomes). However, this will only be possible if our ABI/SBIRT community use it and we invite feedback to support its use.

### O29 Parent and pediatrician perspectives on communicating adolescent substance use risk: implications for screening and brief intervention

#### Stacy Sterling^1^*, Pat Aussem^2^, Sean Clarkin^2^, Christina Grijalva^1^, Felicia Chi

##### ^1^Kaiser Permanente Northern California Division of Research, Oakland, CA, USA; ^2^Partnership to End Addiction, New York, USA

###### Correspondence: Stacy Sterling (Stacy.a.sterling@kp.org)

*Addiction Science & Clinical Practice* 2022,**17(Suppl 1)**: O29

**Background: **Clinicians' ability to predict which youth are at highest risk of substance use problems is limited. While use of predictive analytics in healthcare has grown, predictive models of adolescent substance use problems have not emerged. Using machine learning and data from > 40,000 adolescents, we developed such models, however it is important to identify acceptable strategies for disseminating this risk information to healthcare providers and families for identifying those at greatest risk and targeting prevention and early intervention services.

**Methods: **In order to identify strategies for disseminating information on risk factors from the predictive models, we conducted 9 parent focus groups, of "general population," Black, Hispanic, and Asian parents, parents of younger children, and those with identified risk; and 3 pediatrician groups with physicians working in urban, rural and suburban communities. Groups explored how substance use fit into current parental concerns, perceptions of the parent-pediatrician relationship; roles in prevention and intervention; and strategies for disseminating information about the risk factors identified through the predictive models, including a draft online assessment tool. Pediatrician groups explored the utility of the predictive model for clinical practice. Data were analyzed using a content analysis approach.

**Results: **Substance use is not many parents' highest concern, and they do not know when to address it, or where to turn for guidance. We found a large disconnect between parents and pediatricians' views of the pediatricians' role in screening and prevention of problems. Parents believe pediatricians do little screening, while pediatricians report monitoring "behind the scenes," and discussed confidentiality challenges. Parents were receptive to a tool that allows them determine if their child is at heightened risk.

**Conclusions: **Evidence-based information on risk could help parents and providers intervene to ameliorate risk and prevent substance use problems, if compelling, effective formats for delivery can be identified.

**Trial Registration**: ClinicalTrials.org NCT02408952.

### O30 Predictors and moderators of substance use and sexual risk behaviors of adolescents after brief interventions

#### Anjalee Sharma^1^, Kristi Dusek^1^, Shannon G. Mitchell ^1^, Robert P. Schwartz^1,2^, Courtney D. Nordeck^1^, Kevin E. O. Grady^1,2^, Jan Gryczynski^1^

##### ^1^Friends Research Institute, USA; ^2^Department of Psychiatry, University of Maryland, Baltimore, USA

*Addiction Science & Clinical Practice* 2022,**17(Suppl 1)**: O30

**Background: **School-based health centers (SBHCs) expand access to healthcare services for adolescents and are promising settings for brief interventions (BI). Adolescents may differ in their risk behavior patterns and responsiveness to different types of BI delivery based on individual and social network characteristics. The current study examined potential predictors and moderators of substance use and sex risk behavior trajectories over time, and the extent to which such characteristics are associated with differential response to BI delivery approaches.

**Methods: **This is a secondary analysis of data from a randomized trial of computer- versus nurse-practitioner-delivered BI (CBI vs. NBI) with 300 SBHC patients, ages 14–18. Assessments were conducted pre-BI, and at 3- and 6-month follow-up. Associations between risk behaviors (days of alcohol use, marijuana use, unprotected sex, and sex while intoxicated) and variables of interest (gender, age, severity of substance use problems [CRAFFT score], friend’s substance use, friend’s sex behaviors) were examined using mixed effects negative binomial and logistic regressions with contrasts and individual/social factor X time X BI interactions to examine possible moderation.

**Results: **Older age and engagement in risk behaviors among friend networks had independent associations with the risk behaviors examined across time points (ps < 0.01). Neither substance use (alcohol and marijuana) nor sexual risk behavior trajectories differed on the basis of the variables examined (all ps > 0.05). Moreover, there were no significant differences in the effects of nurse practitioner- vs. computer-delivered BI over time on the basis of the potential moderators examined (all ps > 0.05).

**Conclusion: **Certain adolescent baseline characteristics (particularly age and friends risk behaviors) were associated with adolescents risk behaviors across all time points. Neither individual nor social factors were significantly associated with trajectories of risk behaviors over time, and none moderated the effectiveness of BI delivery approaches.

### O31 Psychological distress in patients with Substance use disorder following COVID-19 lockdown in India

#### Sidharth Arya^1^, Rajiv Gupta^1^, Sunila Rathee^2^, Vinay Kumar^2^

##### ^1^Institute of Mental Health, Pt BDS University of Health Sciences, Rohtak, India; ^2^State Drug Dependence Treatment Centre, IMH, Pt. BDS University of Health Sciences, Rohtak, India

*Addiction Science & Clinical Practice* 2022,**17(Suppl 1)**: O31

**Background: **COVID-19 pandemic has been postulated to have detrimental impact on overall mental health of individuals. People with substance use disorders are highly vulnerable given their maladaptive coping. This study was aimed to explore the impact of COVID-19 lockdown on the mental health of substance users.

**Methods: **We reached out to patients attending substance use outpatient services following the sudden imposition of lockdown via telephonically. The response rate was close to 50%. The patients were inquired about their current substance and medication status as well as adjustment, anxiety, depression and quality of life using Brief adjustment scale-6 (BASE-6), State Trait anxiety Inventory (STAI), physical health Questionnaire (PHQ-2) and single item from WHO-qol Bref respectively.

**Results: **A total of 286 patients responded with predominantly male (99%), 25–45 years (53%), using opioids (53%) or alcohol (33%). Significantly High Scores were reported on measures of anxiety, depression and adjustment by patients with current substance use, recent treatment initiation, off medication and frequent craving.

**Conclusion: **Our findings point out that those with recent treatment initiation, current substance use or inadequate management were more likely to suffer from psychological distress due to sudden closure of treatment services. Since COVID-19 is likely to run a long course and further lockdown remains a possibility, patient recently initiated on treatment for drug dependence may benefit with brief interventions and psychoeducation programs related to coping with COVID-19.

### O32 Readiness to change in men with risk and harmful alcohol use: effect of brief group intervention

#### Erika G. L. Ramírez*, Divane de Vargas, Stephen Strobbe

##### Nursing School of São Paulo University, São Paulo, Brazil

###### Correspondence E-mail: Erika G. L. Ramírez (egleonr@usp.br)

*Addiction Science & Clinical Practice* 2022,**17(Suppl 1)**: O32

**Background: **The prevalence of problematic alcohol use in men is higher than women at the world. In the region of the Americas, this is evidenced by an increase of 4.6% to 13% in women and 17.9% to 29.4% in men between 2013 and 2015. Due to that, this population showed high risk to health and social consequences of the harmful alcohol use and it is necessary to invest in strategies that help them to change the risk behavior. Brief group intervention can be an effective strategy in the change process in persons who use alcohol at risk or harmful, so this study aims to assess the effect of IBG on readiness for change and its relationship with the pattern of alcohol use in men.

**Materials and methods: **Randomized clinical trial, conducted in a primary health care unit in São Paulo (Brazil). 112 men were randomized in the experimental group (n = 55) and in the control group (n = 57). The Readiness to Change Ruler (RTC) was used to assess the RTC, and the Alcohol Use Disorders Identification Test (AUDIT) was used to assess the alcohol use. The experimental group received a brief group intervention; the control group was instructed to continue with the unit's standard care. The data were analyzed using the Generalized Equations Estimating method, Pearson's correlation and the square root of the coefficient of determination (R^2^) of a mixed effects model with RPM predicting AUDIT.

**Results: **Significant difference was observed between the experimental (GE) and control (CG) groups overtime. The correlation between readiness to change and pattern of use over time was reasonable.

**Conclusion: **BGI was shown to be effective in increasing readiness for change when compared to the control group. There was a correlation between readiness for change and a decrease in the pattern of alcohol use after BGI.

**Trial Registration: **Registered in the Brazilian Clinical Trials Registry/Registro Brasileiro de Ensaios Clínicos (ReBEC). REBEC registration number: RBR-6zk87m-UTN number: U1111-1205-8313.

### O33 Reducing women alcohol use: experience of brief interventions’ pilot study at a primary health care service in Brazil

#### Divane de Vargas, Talita D. Ponce, Erika G. L. Ramirez

##### ^1^Nursing School of São Paulo University, São Paulo, Brazil

*Addiction Science & Clinical Practice* 2022,**17(Suppl 1)**: O33

**Background: **Brief interventions (BI) have been suggested for preventing alcohol use by women in the primary health care (PHC). This study aimed to assess the IB feasibility and estimate parameters to design a future study about the effectiveness of BI in reducing the alcohol use among women with hazardous and harmful alcohol use in a PHC in Brazil.

**Methods: **Two-arm pilot randomized trial carried out with 44 women with hazardous or harmful alcohol use and were aged 18 or over. The intervention Group (IG) received the BI in a single session lasting 20 to 30 min. The control Group (CG) received five minutes of Brief Advice. The pattern of alcohol use was assessed by Alcohol Use Disorders Identification Test (AUDIT) and frequency and quantitate of alcohol consumed in the previous month were assessed at baseline and the first and third months of follow-up in both groups.

**Results: **There was a decrease in the AUDIT score in both groups, at baseline (IG 12.89, CG 10.64), in the 1st month (IG 12.78 p = 0.9; CG 7.9 p = 0.01) and in the 3rd month (IG 10.11 p = 0.13; CG 7.09 p < 0.01). The IG maintained the pattern of alcohol use after BI, there was a reduction on quantitate of alcohol consumption compared with baseline (p < 0.01).

**Conclusion: **The Brief intervention was feasible with modifications. The BI delivered in the context of a primary health care service in Brazil has shown potential to reduce women pattern of alcohol consumption.

### O34 Referral for brief intervention for problematic alcohol use (PAU) among transgender females-what do community members think? a qualitative study

#### Sree T. Sucharitha^1^*, R. Pradeep^2^, I. Kannan^1^, M. Nila 

##### ^1^Tagore Medical College Hospital, India; ^2^Department of Psychiatry, Madha Medical College, Chennai, India

###### Correspondence: Sree T. Sucharitha (sucharithat2@tagoremch.com)

*Addiction Science & Clinical Practice* 2022,**17(Suppl 1)**: O34

**Background: **Problematic alcohol use (PAU) among LGBTQ + is increasingly recognized as inadequately addressed public health problem with multiple i.e., biological, clinical, psychosocial sequelae. 'Minority stress', discrimination, victimization, harassment, mental health, targeted marketing, low socio-economic status and personal stress were identified to be associated with high substance use as a coping mechanism. Enacted and felt stigma, isolation among mixed groups has been significant players in excluding the members of the LGBTQ + community from treatment settings. To the best of our knowledge, studies exploring the community members' perspectives in PAU are rare to find among Indian published literature. The aim of the present study was to qualitatively explore the perspectives about brief treatment interventions for PAU among Transgender females in Chennai.

**Methods: **Semi-structured personal interviews were conducted (15) and thematic content analysis was used to analyse qualitative data and themes were documented.

**Results: **Four major themes were identified with PAU and these were found clustered around the social determinants of transgendered lifestyle: heightened stress associated with sexual identity, family rejection and non-acceptance, stigma and discrimination by society, inhumane treatment leading them to resort to begging and menial jobs. However, all the interviewees expressed a strong desire to quit alcohol completely with medical assistance in the form of prescription drugs or restrict their consumption to social occasions only.

**Conclusions: **Transgender female participants in this study were aware of PAU and were strongly motivated to quit problematic alcohol use. Majority expressed a felt need for residential alcohol de-addiction assistance programs. According to them, the brief interventions will not be effective due to the strong pull of hard triggers and lack of social support systems like family and friends who motivate them to quit unlike the general population. Inclusive involvement of our study findings in intervention design for transgenders will support their health needs.

### O35 Results from a Danish feasibility study of a new innovation for screening and brief intervention for alcohol problems in primary care: The 15-method

#### Peter N. Schøler^1^, Jens Søndergaard^2^, Sverre Barfod^2^, Anette S. Nielsen^1^

##### ^1^Unit for Clinical Alcohol Research, Clinical Institute, The University of Southern Denmark, Odense, Denmark; ^2^Unit of General Practice, Department of Public Health, The University of Southern Denmark, Odense, Denmark

*Addiction Science & Clinical Practice* 2022,**17(Suppl 1)**: O35

**Background: **The 15-method is a new brief intervention tool for alcohol problems in primary care which have shown promising results in Sweden for mild to moderate alcohol use disorders. The present study evaluated the 15-methods usability, organizational integration and overall implementation feasibility in Danish general practice (GP) in preparation for a large-scale evaluation of the methods effectiveness in identifying and treating alcohol problems in GP. 

**Methods: **Five general practices in the Central and Southern Region of Denmark participated: seven doctors, five nurses. Participants received a half day of training in the 15-method. Testing of implementation strategies and overall applicability ran for two months. A focus group interview and two individual interviews with the participating doctors along with five individual patient interviews were conducted after the study period. Data is currently being analyzed. 

**Results: **Results indicate implementation of the 15-method is feasible in Danish general practice. The healthcare professionals and patients were positive about the method and its possibilities. The method was considered a new patient centered treatment offer and provided structure to a challenging topic. An interdisciplinary approach was much welcomed. Results indicate the method is ready for large scale evaluation. 

**Conclusions: **Implementation of the 15-method is considered feasible in Danish general practice and large-scale evaluation is currently being planned. Final results from the present feasibility study along with an overview of the large-scale evaluation will be presented at the conference.

### O36 SBIRT as a tool for prevention and reducing risky substance use behaviours among youths in Zaria, Kaduna state, Nigeria

#### Fatima A. Popoola^1^, Olibamoyo Olushola^2^, Lasebikan Victor^3^, Ola B Adeyemi^2^

##### ^1^National Drug Law Enforcement Agency, Lagos, Nigeria; ^2^Department of Behavioural Medicine, Lagos State University College of Medicine Ikeja, Lagos, Nigeria; ^3^Department of Psychiatry, College of Medicine, University of Ibadan, Ibadan, Nigeria

*Addiction Science & Clinical Practice* 2022,**17(Suppl 1)**: O36

**Background: **There is a paucity of research on the effectiveness of SBIRT in a non-medical setting with populations referred majorly from the criminal justice system in low resource settings. These populations have high rates of substance use but have limited access to interventions.

**Materials and methods: **A simple random sampling technique was used for this research, 75 participants were selected from a pool of 150 users, referred from criminal justice, place of work, and schools. Using the Alcohol, Smoking, and Substance Involvement Screening Test (ASSIST), the intervention assessed the risk level for illicit drug use by participants and provided those who were at low or medium risk with a brief intervention and referred those at high risk to intensive treatment. Readiness to Change Questionnaire (RCQ) was used to assess the level of motivation of participants. The intervention was given at baseline and 3 months following baseline intervention. Assessments were carried out at baseline and 6 months. Using interviews and records data from baseline and 6-month, analyses compared the differences. All analyses were set at 95% CI, p < 0.05, and were carried out by SPSS 22.0.

**Results: **We found that the risk of harmful use of cannabis, prescription opioids, and sedatives reduced significantly between baseline and 6-months so also were their mean ASSIST scores. Furthermore, participants have a statistically significant better level of motivation to stop the use of cannabis, prescription opioids, and sedatives between baseline and 6-months. Although participants have reduced risks of harmful use of solvent, the differences between baseline and 6-months were not significant.

**Discussion: **This study has illustrated that the use of screening and the administration of brief interventions for reducing the harmful risk and improving the level of motivation to stop substance use in a non-medical setting can be feasible and effective.

### O37 SBIRT on reducing the risky and harmful consumption of alcohol, in workers of an urban transports service

#### Tereza Barroso, Lícia Teixeira, Daniela Pinto, Fernanda C Branco

##### Nursing School Coimbra/Health Sciences Research Unit: Nursing, Portugal

*Addiction Science & Clinical Practice* 2022,**17(Suppl 1)**: O37

**Background: **Alcohol consumption is an influential factor for decreasing worker performance, increasing the risk of accidents, errors, delays and absenteeism, which can culminate in unemployment. A work environment of great effort, stress and low wages can lead to alcohol consumption, as the person seeks in the effects of alcohol a way to mitigate mental suffering and emotional overload. The objective is to assess whether Brief Interventions have a positive effect on reducing the risky and harmful consumption of alcohol, in workers of an urban transports service.

**Methods: **Level IV design study, quasi-experimental nature, with assessment before and after with a single group. The Screening and Brief Intervention and Referral to Treatment protocol was implemented and the Alcohol Use Disorders Identification Test (AUDIT) was used as an assessment tool. Interventions were performed using the score obtained in this. Evaluation was carried out at follow-up, 4 months later.

**Results: **Of the 45 participants in the first intervention, 39 of these made up the sample, with 7 people presenting a risk level of alcohol consumption and the remaining 32 a level of low risk consumption. At the follow-up, 5 participants reduced their level of risk of alcohol consumption and 34 maintained the same level (since they were already at the low risk level).

**Conclusion: **The results show a positive effect in reducing the alcohol consumption level of risk associated with the intervention performed. Future studies should be conducted with a bigger sample, with a control group and increasing the time between the implementation and the evaluation.

### O38 Screening and brief interventions for excessive alcohol use during pregnancy: practices among U.S. primary care providers, DocStyles 2019

#### Caitlin Green^1^*, Youngjoo Park^1,2^, Nisha George^1,3^, Clark H. Denny^1^, Mary K. Weber^1^, Shin Y. Kim^1^

##### ^1^Division of Birth Defects and Infant Disorders, Centers for Disease Control and Prevention, USA (CDC); ^2^Oak Ridge Institute for Science and Education (ORISE), Oak Ridge, USA; ^3^Eagle Global Scientific, Emory University, Rollins School of Public Health, Atlanta, USA

###### Correspondence: Caitlin Green (lkj8@cdc.gov)

*Addiction Science & Clinical Practice* 2022,**17(Suppl 1)**: O38

**Background: **Prenatal alcohol exposure is a leading preventable cause of birth defects and developmental disabilities in the United States. However, a recent study found that more than 10% of pregnant women reported drinking alcohol within the past 30 days. The US Preventive Services Task Force recommends screening and brief intervention to reduce excessive alcohol use among adults, including pregnant women, for whom any use is considered excessive. This study examines alcohol screening and brief intervention (SBI) practices among primary care providers.

**Methods: **A cross-sectional analysis on self-reported 2019 DocStyles data was conducted to examine alcohol SBI practices by a convenience sample of 1,500 primary care providers (family practitioners, internists, obstetricians/gynecologists, nurse practitioners, and physician assistants) registered to complete an online medical survey. Descriptive statistics were used to explore confidence in conducting alcohol SBI for pregnant women, identify most commonly used screening tools for pregnant women, and document brief intervention practices in medical records.

**Results: **For their pregnant patients, less than half of providers reported feeling very confident in identifying excessive alcohol use (43.5%) and conducting brief interventions (32.5%); 9.5% stated they do not screen for excessive alcohol use. The two most reported screening methods were a single question about the number of days per week they have at least one alcoholic drink (56.9%) and the CAGE (Cut down, Annoyed, Guilty, Eye-opener; 54.7%). Most providers documented brief intervention practices using the electronic health record either in notes (51.7%) or a designated space (50.7%).

**Conclusion: **Less than half of the primary care providers in this sample feel very confident in conducting alcohol SBI with their pregnant patients. Increased training about alcohol SBI, tailored to pregnant women, may increase provider’s™ confidence and uptake of this intervention, and offer opportunities to prevent adverse outcomes from prenatal alcohol use.

### O39 Socioeconomic differences in population beliefs and attitudes regarding alcohol conversations in routine healthcare in England, Netherlands, Norway, and Sweden

#### Torgeir G. Lid, Nadine Karlsson, Janna Skagerström, Amy O'Donnell, Latifa Abidi, Kristin Thomas, Per Nilsen

##### Stavanger University Hospital, Stavanger, Norway

*Addiction Science & Clinical Practice* 2022,**17(Suppl 1)**: O39

**Background: **There is little knowledge of how socioeconomic status influences beliefs and attitude towards delivery of alcohol conversations in healthcare settings. Previous published studies from the research group have examined correlates related to beliefs and attitudes separately in England, Netherlands, Norway, and Sweden. In England, we found those in lower socio-economic groups were less supportive of alcohol conversations in routine healthcare. This study will combine data from all four countries and use a novel way to measure beliefs and attitudes as indexes derived by multivariate to detect socioeconomic differences between countries. Aim to investigate socioeconomic differences in beliefs and attitudes of the population regarding alcohol conversations in routine healthcare in England, Netherlands, Norway and Sweden.

**Methods: **Population-based cross-sectional surveys of men and women aged 16+ were conducted in England (n = 3499), Netherlands (n = 2173), Norway (n = 1208), and Sweden (n = 3000). Two composite dimensions of beliefs and attitudes of the population regarding alcohol conversations in healthcare will be derived through a factor analysis using the data of the four countries. Logistic regression analysis will be used to examine associations between high-level scores of the beliefs and attitudes dimensions and demographic, personal and alcohol correlates. 

**Results: **A preliminary factor analysis was performed with the Swedish and Norwegian populations and allowed us to derive the 2 following different factors: (I) attitude towards discussing alcohol with health care providers and (II) trust and confidence towards health care. Preliminary results showed that "trust" was an important predictor of beliefs and attitudes and is presumably related to overall level of trust in healthcare and in health inequalities.

**Conclusion: **This will provide knowledge from a patient perspective how to target disadvantaged groups and will contribute to increased knowledge that can be used to reduce socioeconomic differences in access to prevention in healthcare.

### O40 Targeting at-risk alcohol use as part of a proactive automatized lifestyle intervention in general hospital patients: Investigation of need

#### Prof J. Freyer-Adam^1^, Filipa Krolo^1^, Anika Tiede^1^, Kristian Krause^1^, Ulrich John^1,2^

##### ^1^University Medicine Greifswald, Institute for Medical Psychology, Greifswald, Germany; ^2^German Center for Cardiovascular Research, Site Greifswald, Greifswald, Germany

*Addiction Science & Clinical Practice* 2022,**17(Suppl 1)**: O40

**Background: **The majority of chronic diseases are preventable. Among modifiable causes, at-risk alcohol use and other behavioral health risk factors (HRFs), foremost tobacco smoking, physical inactivity and unhealthy diet play a crucial role. Co-occurring behavioral HRFs have more than merely additive effects on risk of diseases and mortality. The study aimed to determine the need for comprehensive lifestyle interventions in general hospital in patients with at-risk alcohol use.

**Methods: **Data was collected at four university hospital departments (general surgery, internal medicine, ear-nose-throat, trauma surgery), over a total of 10 weeks in 2020/2021. On four-week days, each 18–64 year old patient admitted on the previous day was proactively approached by study staff, and asked to participate in a survey on health and health behaviors. Of all patients eligible (n = 357), 77.8% participated, and data of n = 256 were analyzable. HRF "alcohol" was determined using the AUDIT-C (≥ 4/5 for women/men). Any tobacco smoking was considered as "smoking". Less than 150 min of moderate physical activity per week were considered as "physical inactivity". Less than five portions of vegetable/ fruit per day were used as indicator for "unhealthy diet". Patients were asked whether diagnoses for any of four chronic disease groups (cancer, cardio-vascular disease, respiratory disease, diabetes) exist.

**Results: **Of all patients screened, 25.5% were at-risk alcohol users (n = 65). Of these, 92.3% (n = 60) reported additional HRFs: n = 23 one, n = 26 two and n = 11 three. Low fruit and vegetable intake was reported by 89.2%, smoking by 46.2%, and physical inactivity by 30.8% of all at-risk alcohol users. Almost half (47.7%) reported at least on chronic disease.

**Conclusions: **The need for systematic screening and comprehensive lifestyle intervention is high. It may serve primary and secondary prevention purposes, by preventing the onset of or by improving the treatment of chronic diseases. Funding: German Cancer Aid (70110543).

### O41 Treatment needs of patients with severe alcohol use disorders: A qualitative study

#### Venkata L. Narasimha^1^, B. A. Aravind^2^, Ramana Tadepalli^3^, Arun Kandasamy^4^, Pratima Murthy^4^*

##### ^1^All India Institute of Medical Sciences (AIIMS), Deoghar, Jharkhand, India; ^2^Department of Epidemiology, National Institute of Mental Health and Neuro Sciences (NIMHANS), Bengaluru, India; ^3^Real Estate Research Initiative, Indian Institute of Management, Bengaluru, India; ^4^Department of Psychiatry, National Institute of Mental Health and Neuro Sciences (NIMHANS), Bengaluru, India

###### Correspondence: Pratima Murthy (pratimamurthy@gmail.com)

*Addiction Science & Clinical Practice* 2022,**17(Suppl 1)**: O41

**Background: **Long-term treatment outcomes remain poor among patients with severe Alcohol Use Disorders (AUDs). Identifying and addressing treatment needs can improve outcomes.

**Methods: **A qualitative study design was used to assess the treatment-related needs of patients with alcohol use disorders admitted to a tertiary care treatment center. A semi-structured questionnaire was developed with anchor questions based on the literature review and key informant interviews. All the interviews were audio-recorded, transcribed, and color-coded manually. Thematic inductive analysis was done, the codes were reviewed by two reviewers, and themes and subthemes generated.

**Results: **Among the 15 patients interviewed, all the patients had severe alcohol use disorder (100%), were married (100%), mostly males (86.6%), and more than half below the poverty line (53.4%); with a mean age of 41.1 years (s.d = 9.5). Four major themes were identified i) individual-related needs ii) family-related needs iii) hospital-related needs iv) community-related needs. Among individual needs, medications, and related information; psychological and occupational needs were uppermost concerns. Family needs were predominantly support, psychoeducation, and reducing conflict. Hospital needs focused on different services in the inpatient and outpatient; characteristics of the treatment environment, behavior of treating team and scheduling daily activities during treatment. In the community, perceived needs included treatment- accessibility, availability, affordability, alternatives, and awareness; reducing barriers and alcohol availability were prominent.

**Conclusion**:The treatment needs of the patients with AUDs are diverse. While individual and family needs highlight the important aspects of interventions, hospital and community-based needs underscore the structural and functional aspects of treatment.

### O42 Training common factors in Addictions Treatment: Teaching frontline providers to do what they already do, but really, really well

#### Molly Magill*

##### Centre for Addiction Studies, Brown University Public Health, Providence, USA

###### Correspondence: Molly Magill (molly_magill@brown.edu)

*Addiction Science & Clinical Practice* 2022,**17(Suppl 1)**: O42

**Background: **Dissemination of evidence-based therapies (EBTs) to clinical settings continues to be a challenge. Not only is the evidence sparse for selecting a single, best EBT for widespread dissemination and implementation (D&I), but the paradigm that argues for the application of a packaged (i.e., manualized) EBT also does not accommodate the many complexities of frontline care.

**Methods: **The current project has undertaken a systematic review and qualitative content analysis of over 100 evidence-based sources in the addictions, behavioral and mental health, medicine, as well as a range of other disciplines (e.g., social psychology). These sources include government-issued practice guidelines, literature reviews or other theoretical works from peer-reviewed sources, therapy manuals, and therapy demonstration videos.

**Results: **While addictions was a central focus, sources were transdiagnostic. The goal was to derive clear training guidelines along five topical areas: 1) Developing a working relationship, 2) Goal setting and monitoring, 3) Providing psychoeducation, 4) Providing skills training, and 5) Working with naturalistic support systems. Each topical area, or module, is characterized by a set of principles (i.e., a general understanding or way of being on the part of the provider that is kept in mind when implementing a specific therapeutic practice) and practices (i.e., a more concrete action step or technique used by the provider when delivering specific therapeutic content). This work is content agnostic, and instead focuses of the 'how' of intervention delivery.

**Conclusions: **The overarching goal of this project is to operationalize core processes of addictions therapies that are broadly applicable to a range of provider types, clinical contexts, and thus content foci. Further, the processes targeted are those that most or all providers must engage in, and the emphasis here is engaging in these processes in a manner consistent with best practices and EBT.

### O43 Young adult substance use, mental health and medical outcomes and healthcare use associated with screening, brief intervention and referral to treatment in pediatric primary care

#### Stacy Sterling*, Sujaya Parthasarathy, Ashley Jones, Constance Weisner, Verena Metz, Lauren Hartman, Katrina Saba, Andrea H Kline-Simon

##### Kaiser Permanente Northern California Division of Research, Oakland, CA, USA

###### Correspondence: Stacy Sterling (Stacy.a.sterling@kp.org)

*Addiction Science & Clinical Practice* 2022,**17(Suppl 1)**: O43

**Background: **Most studies on adolescent screening, brief intervention and referral to treatment (SBIRT) have examined shorter-term substance use outcomes. However, SBIRT may also impact comorbidity and health services utilization—an understudied topic. We address this gap by examining effects of SBIRT on substance use, mental health and medical outcomes, and healthcare utilization, over 7 years post-screening.

**Methods: **In a randomized trial sample, we assessed 3 SBIRT modalities: 1) pediatrician-delivered, 2) behavioral clinician-delivered, and 3) usual care. Substance use, psychiatric and medical comorbidity and healthcare utilization were compared between a brief intervention group with access to SBIRT for behavioral health (combined pediatrician and behavioral clinician arms) versus a group without access (Usual Care), over 5 and 7 years.

**Results: **At 5 years, compared to Usual Care, the SBIRT group had fewer substance use (15.0% vs. 20.6%, p < 0.01), alcohol use (3.8% vs. 6.8%, p < 0.01), any drug use (8.4% vs. 11.9%, p < 0.05), and tobacco use (7.7% vs. 10.7%, p < 0.05) disorder diagnoses. At 7 years, the SBIRT group continued to have fewer substance use (19.0% vs. 24.0%, p < 0.05), alcohol use (4.8% vs. 7.8%, p < 0.01), and any drug use (10.6% vs. 13.8%, p < 0.01) disorder diagnoses, compared to Usual Care. At 5 years, a higher percentage of those in the SBIRT group had at least one psychiatry visit (43.5% vs. 34.7%, p < 0.001), and a lower percentage had addiction medicine visits (4.4% vs. 6.8%, p < 0.05) or inpatient hospitalizations (10.3% vs. 14.5%, p < 0.01), compared to Usual Care. At 7 years, a higher percentage of those in the SBIRT group had psychiatry visits (47.4% vs. 39.9%, p < 0.001), and a lower percentage had addiction medicine visits (4.9% vs. 7.8%, p < 0.05) or inpatient hospitalizations (13.3% vs. 18.2%, p < 0.01), compared to Usual Care.

**Conclusions: **Providing SBIRT in pediatric primary care use may have enduring effects on substance use and healthcare utilization.

## Poster Presentation Abstracts

### P1 A multi-stakeholder approach to developing outcome measures to evaluate an initiative promoting management of unhealthy alcohol use in primary care

#### Shana Sandberg^1^, Tracy McPherson^1^*, Adrienne Call^1^, Jenna T. Sirkin^1^, Alexandria Figueroa^1^, Marian James^2^, Brent Sandmeyer^2^

##### ^1^NORC at the University of Chicago, Chicago, USA; ^2^ U.S. Agency for Healthcare Research and Quality, Bethesda, USA

###### Correspondence: Tracy McPherson (Mcpherson-tracy@norc.org)

*Addiction Science & Clinical Practice* 2022,**17(Suppl 1)**: P1

**Background: **Prior to the COVID-19 pandemic, excessive alcohol use had been the third leading cause of preventable death in the United States, affecting nearly a third of adults but remaining largely unaddressed in primary care (PC) settings. In 2019 the U.S. Agency for Healthcare Research and Quality (AHRQ) launched an initiative to fund six grantees to disseminate evidence-based approaches managing UAU in 70 PC practices. Given the lack of national consensus on outcome measures to assess rates of screening, brief intervention, and medication assisted therapy (MAT), we took a multi-stakeholder approach to develop a novel measure set for the cross-grantee evaluation of this initiative. We describe strategies used in the initiative's first year to develop measures for use in the mixed-methods evaluation.

**Methods: **We designed a collaborative approach to systematically gather crucial input on measure concepts, relevance, and feasibility from the six grantees and AHRQ. Three primary strategies defined our approach: individual meetings between independent evaluator and each grantee, a cross-grantee evaluation workgroup to facilitate engagement, and regular calls between the evaluator and AHRQ to clarify aims and resolve issues. Overall, we worked to maintain rigor and cross-grantee comparability while remaining agile and flexible in our design.

**Results: **Collectively, we developed six novel outcome measures for PC practices to document and evaluate the target population and number and percent of those receiving screening, brief intervention, MAT initiation, and specialty care referrals. These measures are intentionally designed to accommodate variation in screening tools, grantee interventions, and documentation and reporting (e.g., paper chart abstraction, electronic health records). We highlight overall strengths and limitations of these measures and the degree of alignment with other measures currently in use.

**Conclusions: **Seeking input from diverse stakeholders can aid in developing outcome measures to evaluate an initiative designed to promote management of UAU in PC.

### P2 A psychosocial program for the prevention and mitigation of the consumption of psychoactive substances in young people of Colombia

#### Slyn T. R. Vega, Liliana M. G. Vega, Marco A. M. Gómez*

##### Psicologa, Universidad Nacional Abierta y a Distancia, Bogotá, Colombia

###### Correspondence: Marco A. M. Gómez (marco.marquez@unad.edu.co)

*Addiction Science & Clinical Practice* 2022,**17(Suppl 1)**: P2

**Background: **The consumption of psychoactive substances (SPA) is a worldwide public health problem, and with repercussions at the individual, family, educational, social and community levels, which is why the general objective of this program is to prevent and mitigate the consumption of psychoactive substances based on the guidelines of psychosocial programs, specifically those focused on life skills, with a comprehensive and complex understanding of the problem, and which is based on the development of certain skills in childhood and Adolescence allow to face in a positive and adequate way the different difficulties of daily life and avoid the interest with the consumption of SPA.

**Methods: **To achieve this, the development of an observational-descriptive study with a phase of psychosocial intervention begins. The population was 755 secondary school students from the sixth to eleventh grades of the Agustina Ferro educational institution in the municipality of Ocaña, the sample was randomly made up of 255 students who were administered an online questionnaire on rapid identification of consumption of SPA.

**Results and conclusions:** According to the results, the study group is chosen, a ninth grade course, made up of 36 students who develop a life skills test before starting the implementation of the program, based on the results it is designed a SPA consumption prevention and mitigation program, based on the life skills training model, and with an implementation methodology through participatory and interactive teaching strategies, seeking its results to prevent new cases of consumption SPA, strengthen life skills in students that translate into a better coexistence school and family giants, a more critical and conscious attitude of decisions and behaviors, from a double action, through direct work with students as the object of said intervention, with teachers and parents as preventive agents in their closest environment.

### P3 Barriers and facilitators of the use of alcohol screening and brief intervention within the Accident and Emergency and Urgent Care Centre healthcare environment: a systematic review

#### Christopher Moat^1^, Professor Newbury-Birch^1^, Katie Haighton^2^, Maggie Leese^1^

##### ^1^Teesside University, Middlesbrough, United Kingdom; ^2^Northumbria University, Newcastle upon Tyne, United Kingdom

*Addiction Science & Clinical Practice* 2022,**17(Suppl 1)**: P3

**Background: **Alcohol screening and brief interventions are routinely adopted in primary care environments with the aim of reducing alcohol consumption and related harm in hazardous drinkers who are not currently receiving help. This review aimed to investigate the barriers and facilitators in relation to alcohol screening and brief interventions for adult service users presenting to Accident and Emergency and Urgent Care centres. The doctoral research which this systematic review contributes to focuses on exploring the feasibility of carrying out alcohol screening and brief interventions by Paramedics, in the pre-hospital environment. NHS Ambulance service staff do not currently undertake any screening activities with this patient group. 

**Methods: **A search of medical and social sciences databases was undertaken to locate qualitative studies using methods such as semi-structured and in-depth interviews, focus groups and ethnographical work, to explore the barriers and facilitators for adults in the general population. Surveys which included open-ended questions were also included. Thematic analysis of the transcripts was undertaken and recommendations for a future pilot study were made.

**Results: **Findings from 39 results aligned with and reinforced known barriers and facilitators. Analysis of the data distinctively considered a variety of roles and settings of the professional staff undertaking alcohol screening and brief interventions and acknowledged the thoughts and feelings of service users. Themes which emerged included, training needs and knowledge of staff, staff attitudes, patient issues, lack of time, issues with referral and the location where the screening or intervention is undertaken. 

**Conclusions: **This study has identified barriers and facilitators to successful implementation in a variety of clinical settings, adequate training, resources, and staff motivation were identified as both barriers and facilitators to effective implementation. Further research, through my doctoral research will evaluate the feasibility of carrying out alcohol screening and brief interventions by Paramedics, in the pre-hospital environment.

### P4 Brief intervention for older adults (BIO) delivered by non-specialist community health workers to reduce at-risk drinking in primary care

#### Tassiane C. S. de Paula, Camila Chagas, Cleusa P. Ferri

##### Universidade Federal de São Paulo (UNIFESP), São Paulo, Brazil

*Addiction Science & Clinical Practice* 2022,**17(Suppl 1)**: P4

**Background: **Evidence suggests that brief interventions are effective in reducing alcohol consumption among the adult population. However, the effectiveness of these interventions when delivered specifically to older adults and by community health workers (non-specialists) in a primary health care setting still is unknown.

**Methods: **The Brief Intervention for Older Adults (BIO) is a 10 to 15 min intervention designed to change behavior and includes seven components: 1) feedback; 2) identification; 3) information; 4) reflection; 5) normative; 6) construction; 7) booklet and to be delivered by community health workers (CHW). For this it was developed: a customized teaching manual on the intervention; a script that guides the CHW; and an educational booklet addressing alcohol and aging information. This intervention was designed to be delivered in primary care settings in the Brazilian public health system and to be conducted in an everyday environment. The BIO is a simple, fast, low-cost intervention that could be translated and easily applied by non-specialist professionals working in low-resource settings worldwide. The clinical trial to test the effectiveness of the BIO was approved by Brazilian Clinical Trials Registry—REBEC (nº RBR-8rcxkk) and the protocol of this controlled trial to test the effectiveness of BIO was written and will be published soon. This clinical trial had been started in January 2020, but it was suspended in March 2020 due to the COVID pandemic, in this period it was carried out an initial recruitment considered a pilot study, which allowed to assess the feasibility and acceptance by the participants with positive results. This complete trial will be carried out after the pandemic, when possible.

### P5 Brief intervention in Brazilian workers with an adult AUDIT score of 8 or higher

#### Sandra R. C. Ayub^1^*, Raul A. Martins^2^

##### ^1^FATEC Catanduva—Centro Paula Souza, Catanduva, Brazil; ^2^Universidade Estadual Paulista, São Paulo, Brazil

###### Correspondence: Sandra R. C. Ayub (sandrachalela@gmail.com)

*Addiction Science & Clinical Practice* 2022,**17(Suppl 1)**: P5

**Background: **Alcohol abuse is considered a public health problem, being the third cause of absenteeism in the workplace and the eighth cause of disability benefits granted by Social Security in Brazil. The worker performance and the work environment are affected by the consequences of alcohol abuse, causing a decrease in productivity and quality of work, absences, changes in personal habits, bad working relationships, work accidents, among others. The objective of this research was to identify the pattern of alcohol consumption in workers and then apply the Brief Intervention (BI) in those who presented a pattern of excessive drinking, aiming at withdrawal or at least a decrease in consumption.

**Methods: **This research was exploratory, descriptive, quasi-experimental, and carried out in 4 stages: initial screening, interview, BI, and follow-up sessions. In the first stage, 230 workers took part, from whom 34 percent reached an AUDIT score of eight or higher (classified as positive). In the second stage, these results were confirmed with the Alcohol Dependence Scale (ADS) and the workers were divided into: experimental group (43 workers) and control group (28 workers). In the third stage, the BI was applied only to the experimental group. In the fourth stage, AUDIT was applied after 3 and also 6 months of BI to both groups for comparison.

**Results: **The results showed that BI was effective with workers, both in the first and second follow-up sessions. In all follow-up sessions, the data showed that the experimental group decreased their amount of drinking. The control group also showed a decrease in the intake of alcoholic drinks, but not in the same proportion as the experimental group.

**Conclusion: **We believe these results can contribute to the implementation of programs aimed at the employees' health using the BI, which is easy to apply, quick, inexpensive and effective.

### P6 Cannabis use, comorbidities, and polypharmacy among older adults receiving care in a large urban healthcare system 2019–2020

#### Marjan Javanbakht^1^, Sae Takada^2^, Whitney Akabike^3^, Lillian Gelberg^3^*, Steven Shoptaw^3^

##### ^1^UCLA Fielding School of Public Health, Los Angeles, CA, USA.; ^2^ UCLA David Geffen School of Medicine, Department of Internal Medicine, Los Angeles, CA; ^3^ UCLA David Geffen School of Medicine, Department of Family Medicine, Los Angeles, CA

###### Correspondence: Lillian Gelberg (lgelberg@mednet.ucla.edu)

*Addiction Science & Clinical Practice* 2022,**17(Suppl 1)**: P6

**Background: **The aim is to describe the prevalence of cannabis use and co-use with prescription medications among patients ≥ 50 years of age attending primary care (PC) clinics in a large urban healthcare system in Los Angeles, CA, after legalization of recreational cannabis use.

**Methods: **We used electronic health record (EHR) data from over 60 PC clinics of patients' ≥ 50 years of age who had an annual physical examination between July 2019 and May 2020. Cannabis use was assessed by clinical staff at the time of the visit. We also used EHR data on clinical characteristics including current prescriptions and comorbidities (ICD-10).

**Results: **42,455 patients were included: median age 63 years (range: 50–101), 56% female; 66% identified as white/Caucasian, 10% Asian, 9% Hispanic/Latinx, and 5% black/African American. Current cannabis use was reported by 7.6% and higher than tobacco use (4.0%). Prevalence of cannabis use was higher among those with a current diagnosis of respiratory (9.1% vs. 7.6%; p value = 0.03) or psychiatric condition (9.7% vs. 7.3%; p value < 0.01). Cannabis use was also higher among those prescribed inhaled short-acting beta agonists/anticholinergics (8.6% vs. 7.5%; p value < 0.01), benzodiazepines (10.9% vs. 7.3%; p value < 0.01), antiepileptics (13.6% vs. 7.6%), opioids (12.0% vs. 7.5%; p value < 0.01), or muscle relaxants (10.3% vs. 7.5%; p value < 0.01). After adjusting for age, sex, race/ethnicity, and comorbidities (Charlson Comorbidity Index), those prescribed medications for psychiatric (adjusted OR = 1.5; 95% CI 1.4–1.7), respiratory (adjusted OR = 1.2; 95% CI 1.1–1.3), or neurologic conditions (adjusted OR = 1.4; 95% CI 1.2–1.5) had increased odds of cannabis use compared to those not prescribed these medications.

**Conclusion: **The prevalence of cannabis use among older adults in PC is high and higher among those prescribed medications which may interact with cannabis. Older patients may benefit from routine PC screening for cannabis use and brief advice regarding cannabis use and other prescription medications.

### P7 Correlation between therapist´s competencies and change factors in users

#### Karen I. M. Williams*, Violeta F. Romero

##### Universidad Nacional Autónoma de México, Mexico, Mexico

###### Correspondence: Karen I. M. Williams (5karenmontoyaw@gmail.com)

*Addiction Science & Clinical Practice* 2022,**17(Suppl 1)**: P7

**Background: **The aim of this study was to evaluate the effects of the implementation of brief counseling competencies in addictions by advisers on factors of change in the consumption behavior of the participants attended.

**Methods: **The research included the assessment of adviser´s competencies through direct observation and completion of the Behavioral Assessment Checklist of skills and attitudes, as well as the assessment of motivation, risk perception, stage of change, goal attainment, and levels of general and specific self-efficacy of the participants through self-reporting, The Brief Situational Confidence Questionnaire, and The General Self-Efficacy Scale.

**Results: **The results obtained showed in general an increase in the values of the variables related with the change in the participants with the passage of the intervention sessions. Most of the findings pointed to positive and statistically significant correlations between the implementation of brief counseling competencies by the advisers and the levels of change factors of the participants: (total of competencies and general self-efficacy r = 0.450, p = 0.050, general skills and risk perception r = 0.567, p = 0.011, total of attitudes and general self-efficacy r = 0.513; p = 0.025).

**Conclusions: **These results suggest evidence of the relationship between the health professional's competencies and the effectiveness of brief counseling.

### P8 Efficacy of brief intervention for hazardous and harmful alcohol use in patients with mood disorders: a randomized controlled trial

#### Ankit Sinha, Adarsh Kohli, Debasish Basu, Abhishek Ghosh

##### Department of Psychiatry, Postgraduate Institute of Medical Examination and Research, Chandigarh, India

*Addiction Science & Clinical Practice* 2022,**17(Suppl 1)**: P8

**Background: **Alcohol use disorders and mood disorders are highly co-morbid and individuals are at increased risk of treatment non-compliance and relapse of alcohol use as well as mood episode. Screening and treating alcohol problem is hence very important.

**Methods: **The sample consisted of 84 participants from outpatient of the Department of Psychiatry, PGIMER, Chandigarh, who had hazardous or harmful drinking with Alcohol Use Disorder Identification Test (AUDIT) score of 8 to 19, and an additional diagnosis of mood disorder. Permuted block randomization was used for treatment allocation, with an allocation ratio of 1:1. In addition to usual outpatient care, Intervention group received a brief intervention of 15–20 min and the Control group received general advice only. Outcome was assessed 3 months after the intervention with self-rated Hindi version of the AUDIT and Readiness To Change Questionnaire (RCQ).

**Results: **There was a significant decrease in the mean AUDIT score at the end of follow up in the Intervention group (p = 0.001). Time had a significant effect on the mean AUDIT score (p = 0.005). The change in the AUDIT score remained significant even in the Group X Time interaction (p = 0.001). The effect size was 0.140. A significant difference in the total number of drinking days (Z = − 2.889, p = 0.004), the total number of drinks per drinking day (Z = −2.496, p = 0.013) and the total number of heavy drinking days were observed in the Intervention group (Z = − 2.714, p = 0.007). The mean action score at the end of follow up was significantly more among the Intervention group compared to the Control group (p = 0.032).

**Conclusions: **The present study showed that Brief Intervention is acceptable and feasible in psychiatric out-patient. Future studies should be done in a larger sample, from multiple centres, in both men and women with longer duration and in other psychiatric disorder along with the alcohol issues.

### P9 Integrating Mobile Phone Technology with a Screening and Brief Intervention (SBI) to Prevent Substance Use Dependence during the COVID-19 Pandemic

#### Quynh Vo^1^*, Natalie Martinez^1^, Stephanie Sumstine^1^, Efren Aguilar^2^, Whitney Akabike^2^, Dallas Swendeman^1^, Lillian Gelberg^2^

##### ^1^Department of Psychiatry and Biobehavioral Sciences, David Geffen School of Medicine, University of California, Los Angeles, USA; ^2^Department of Family Medicine, University of California, Los Angeles, USA

###### Correspondence: Quynh Vo (quynhtvo@mednet.ucla.edu)

*Addiction Science & Clinical Practice* 2022,**17(Suppl 1)**: P9

**Background: **The uncertainty caused by the COVID-19 pandemic has seen over 13% of Americans reporting starting or increasing use of illicit substances as a coping mechanism. The Quit Using Drugs Intervention Trial (QUIT) screening and brief intervention (SBI) reduced risky drug use among low-income primary care clinics patients over 3 months.

**Methods: **A new SBI trial, QUIT-Mobile, augments the QUIT intervention by adding mobile phone self-monitoring, weekly automated feedback messages, and dashboards to support telephone coach monitoring of patients' self-monitoring data during coaching sessions. Reduced in-person clinic capacities due to the COVID-19 pandemic and reduced clinic waiting room times led to expansion of telehealth services, which required the QUIT-Mobile team to adapt from in-person screening in clinic waiting rooms to online clinic visit pre-screening and remote patient recruitment, enrollment and assessment. Patients will be randomized into three groups: QUIT-Mobile, Standard-QUIT, and usual care. All participants will receive brief advice from their Primary Care Provider, a video doctor, and health education resources. Patients in the QUIT or QUIT-Mobile arm will be assigned a health coach to monitor patient responses to questionnaires and lead telephone coaching sessions that use motivational interviewing techniques. The QUIT-Mobile arm will receive self-monitoring text-message surveys on a biweekly basis for 6 weeks and on a weekly basis from 6 weeks to 12 months following the brief intervention. Surveys consist of 10 questions regarding drug and alcohol use and cravings, and quality of life. Automated feedback text-messages will use positive reinforcement hypothesized to enhance and sustain the impacts the physician brief advise and telephone health coaching. Participants' experiences will be assessed at 3, 6, and 12-month follow-ups. We hypothesize that those in the QUIT-Mobile group will experience the greatest reduction in drug use.

### P10 Implementing brief intervention in a primary care unit for problems relating to alcohol consumption and other drugs

#### Angela M. Abreu*, Riany MR Brites, Marcia P. César, Juliana R. Dias, Larissa R. Mattos, Débora S. Dias, Angela M. Barros, Reginaldo F. Guimarães

##### Federal University of Rio de Janeiro, Rio de Janeiro, Brazil

###### Correspondence: Angela M. Abreu (angelabreu@globo.com)

*Addiction Science & Clinical Practice* 2022,**17(Suppl 1)**: P10

**Background: **The consumption of alcohol and other drugs is a problem that presents itself to societies around the world and health professionals are not always trained to deal with it, in an appropriate way with the user. The World Health Organization has recommended the application of Brief Interventions, which have shown their effectiveness in this context, in order to reduce problematic substances consumption, especially in Primary Care. Objective: to describe the socio-demographic profile of the users registered at the Service and the adherence to the consultation, by using the Brief Intervention technique.

**Methods: **This is a descriptive research, which brings practical contributions to attend the implementation of the Brief Intervention in a Primary Care Unit, for patients with Problems Related to Alcohol and Other Drugs, in a University Hospital, in Rio de Janeiro / Brazil, applying ASSIST and AUDIT. The sample consisted of 35 patients seen between March and September 2019, based on descriptive statistics with simple frequencies, performed in Excel. It was considered as adherence, patients over three consecutive consultations.

**Results: **In the sample profile there was a higher frequency for males 82.9%, singles 46.0%, age group 40 to 50 years 37.1%, basic education 48.4%, and incomes between 1 to 2 minimum wages 48.4%. Substances used with a higher prevalence in the last three months were alcohol 26.0%, cocaine 23.0%, tobacco 16.0%. Adherence to consultations was 65.7%.

**Conclusions: **The profile is of a population with predominance of males, vulnerable, with low education and income, who use psychoactive substances. There was a good adhesion to the consultation using the protocol of the Brief Intervention. The implementation of a protocol based on Brief Intervention became the guideline for all care in the Service. The study is in progress at the moment.

### P11 Implementing screening, brief intervention and referral to treatment (SBIRT) for drug use in FQHC primary care clinics in the COVID-19 era

#### Lillian Gelberg^1^*, Geoffrey Curran^2^, Stephanie Sumstine^3^, Whitney Akabike^1^, Efren Aguilar^1^, Natalie Martinez^3^, Quynh Vo^3^, Dallas Swendeman^3^

##### ^1^Department of Family Medicine, University of California, Los Angeles, USA; ^2^Departments of Pharmacy Practice and Psychiatry, University of Arkansas Medical Sciences, Arkansas, USA; ^3^Department of Psychiatry, David Geffen School of Medicine, University of California, Los Angeles, USA

###### Correspondence: Lillian Gelberg (lgelberg@mednet.ucla.edu)

*Addiction Science & Clinical Practice* 2022,**17(Suppl 1)**: P11

**Background: **The Quit Using Drugs Intervention Trial (QUIT)-Mobile study is a NIDA-funded Hybrid Type 1 effectiveness-implementation SBIRT aimed at reducing moderate risk drug use in primary care patients in Los Angeles and includes a 12-month follow-up to reflect a model for implementation where patients will be re-screened routinely in annual visits. The study team deployed implementation methods at the formative stages of the study to inform how the intervention might need to be adapted for optimal uptake and sustainability in primary care clinics during the COVID-19 era, especially with telehealth visits.

**Methods: **Weekly Community Advisory Board (CAB) meetings are held with clinic partners. The CAB works collaboratively to adapt the full QUIT-Mobile protocol for telehealth and implementation. Researchers used thematic content analysis of team meeting notes using Dedoose to code for quality improvement, early clues of what QUIT-Mobile investigators and clinicians think about the protocol and how the team is adapting SBIRT for telehealth.

**Results: **Preliminary results include: 1) patients to self-administer computerized drug use screening to become part of the clinic's mandated pre-visit clinic screeners; 2) implementation implications of offering Zoom due to telehealth reimbursement currently only for telephone visits for FQHCs; 3) planning for a Medical Assistant to "hand" PCPs the Clinician Brief Advice Script and Intervention Plan, and exploring cost implications; 4) building in reimbursable screeners to make SUD screening scalable for clinic implementation; and 5) navigating validity of mail-based urine drug screening (UDS).

**Conclusion: **Findings from this analysis show the challenges inherent in shifting procedures to telehealth for patient visits, health education sessions, and mail-based UDS for risky drug users, while navigating clinic challenges during COVID-19. If effective, this study will be integrated into routine primary care as part of behavioral health efforts following recommendations of the Affordable Care Act and the Mental Health Parity Act.

### P12 Patients undergoing treatment for tuberculosis, users of psychoactive substances, who underwent a Brief Intervention from the perspective of adherence to treatment

#### Sonia S. D. E. Santo^1^*, Angela M. Abreu^2^, Luciana F. Portela^3^

##### ^1^Municipal Hospital Raphael De Paula Souza, Rio de Janeiro, Brazil; ^2^Federal University of Rio de Janeiro, Rio de Janeiro, Brazil; Fundação Osvaldo Cruz, Rio de Janeiro, Brazil

###### Correspondence: Sonia S. D. E. Santo (sucessonia@gmail.com)

*Addiction Science & Clinical Practice* 2022,**17(Suppl 1)**: P12

**Background: **The consumption of psychoactive substances by tuberculosis patients results in a public health problem nowadays. The increase in morbidity and mortality, due to tuberculosis and the consumption of psychoactive substances, has a negative impact on the health of society. The objectives are to identify the profile and pattern of consumption of psychoactive substances in tuberculosis patients in the Primary Health Care Unit; to analyze adherence to the treatment of these patients who use psychoactive substances; to carry out the Brief Intervention in these users from the perspective of adherence to the treatment of tuberculosis.

**Methods: **Sectional study, carried out in Primary Care units in the modality of the Family Health Strategy in the city of Rio de Janeiro, in 114 tuberculosis patients, using ASSIST. All patients undergoing tuberculosis treatment were included in the sample. The exposure variable was the consumption of psychoactive substances and the outcome of treatment adherence. The analyzes were treated in the SPSS program, based on chi-square and Fisher's tests, with a value of p ≤ 0.05 being considered.

**Results: **The sample showed the majority of men (71.1%). The mean age was 39.8 years, skin color: 42.1% brown, followed by 31.6% black. They needed to receive a brief intervention for tobacco 33.3%, alcoholic drinks 16.7% cocaine/crack 13.2%. Need to refer for treatment regarding tobacco use 7.9%, followed by 5.3% for alcoholic beverages. Regarding adherence to treatment, there was a prevalence of males (85.0%), forty years of age or older (87.5%), medium/higher education (84.8%), married (86.8%), white (20%), family income up to 1 minimum wage (84.2%).

**Conclusion: **These results demonstrate the importance of a brief intervention in tuberculosis patients who use psychoactive substances, as a care protocol, in order to direct a greater adherence to treatment, in a population with a vulnerable profile.

### P13 Risk perception towards exposure to second-hand smoke from tobacco among antenatal clinic attendees in Primary Health Care (PHC) facilities in Calabar, Nigeria

#### Uchechi C. Onukogu^1^*, Bassey Edet^1^, Owoidoho Udofia^3^, Amarachi C. Kalu^1^, Chidimma A. Anya^2^, Essien Ekpe^1^

##### ^1^Federal Neuro-psychiatric Hospital Calabar, Calabar, Nigeria; ^2^Federal University Gusau Zamfara, Gusau, Nigeria; ^3^Department of Psychiatry, University of Calabar, Calabar, Nigeria

###### Correspondence: Uchechi C. Onukogu (ucheonukogu@gmail.com)

*Addiction Science & Clinical Practice* 2022,**17(Suppl 1)**: P13

**Background: **Globally, use of tobacco is viewed as a serious threat to the health of pregnant women and their unborn children. Maternal Tobacco smoking is associated with different negative birth out comes. However, the extent of passive tobacco smoking and its associated risk in pregnancy is yet to be adequate studied in our region, Calabar Nigeria. Considering the above, the objectives of this study is to determine tobacco use in pregnant women in Calabar Nigeria, and to explore perceived risk of second hand smoke on pregnancy in this population.

**Methods: **Questionnaires were administered to a cross-section of 200 antenatal Clinic attendees randomly selected from Primary Heath Care facilities in Calabar South-south Nigeria. Data were analysed using Statistical Package for Social Sciences version 21. Analysis was both descriptive and inferential at 95% confidence levels.

**Results: **The age of the respondents ranged from 15 to 49 years, with a mean age of 24 years. The prevalence of tobacco smoking in pregnancy was 4% (8/200). About 87% (174/200) admitted negative effect of tobacco smoking both on mother and unborn child. However, there is poor knowledge and perception of risks of second-hand smoking on pregnancy—79.5% (159/200).

**Conclusion: **The prevalence of tobacco smoking among antenatal clinic attendees in Calabar, Nigeria was low. Tobacco use by pregnant mothers is perceived to have higher risk of negative birth outcome as opposed to exposure to second hand smoke. Hence, antenatal heath talks and antismoking programs should include information on maternal and peri-natal diseases/conditions associated with exposure to second-hand tobacco smoking.

### P14 Screening, motivational interviewing, art and music therapy to improve psychosocial wellbeing of participants with hazardous alcohol use in a community based setting

#### Abhishek Chaturvedi^1^, Guruprasad Rao^1^, Samir K Praharaj^2^, K. P. Guruprasad^3^*, Vivek Pais^4^

##### ^1^Department of Biochemistry, Melaka Manipal Medical College (Manipal campus), Manipal Academy of Higher Education, Manipal, India; ^2^Department of Psychiatry, Kasturba Medical College, Manipal, Manipal Academy of Higher Education, Manipal, India; ^3^Department of Ageing Research, Manipal School of Life Sciences, Manipal Academy of Higher Education, Manipal, India; ^4^Akhila Karnataka Jana Jagruthi Vedike, Shree Kshethra Dharmasthala Complex, Belthangady, Karnataka, India

###### Correspondence: K. P. Guruprasad (guruprasad.rao@manipal.edu)

*Addiction Science & Clinical Practice* 2022,**17(Suppl 1)**: P14

**Background: **Art and music, combined with motivational interviewing can be used to alleviate the symptoms of anxiety and depression by providing a relaxing atmosphere where patients stay occupied while harnessing positive energy. The present study explores the use of above modalities to improve the psychosocial wellbeing of hazardous alcohol users in the community settings of South Canara district of Karnataka.

**Methods: **Seventy-four participants reporting for 7-day residential treatment camp at Shree Dharmasthala Manjunatheshwara De-addiction and Research centre, Ujire were enrolled in the study. These participants were interviewed regarding physical and mental well-being (using GAD-7, PHQ-9, PSS and WHOQOL-BREF) and screened for alcohol use disorder using AUDIT. In the 7-day residential treatment camp the group motivational interviewing was done for 60 min every day by social workers. Art and music as a therapy medium was used every day in the evenings for 60 to 90 min. After the 7-day camp, participants were followed up for the total duration of three months from the last day of the camp and assessed again at the end of three months.

**Results: **Twenty-two participants remained abstinent for three month following the treatment camp. The participants who did not attend the follow up had significantly (p < 0.05) higher score for GAD-7 and PHQ-9 at the baseline compared to those who attended the follow up after 3 months. The PSS, GAD-7 and PHQ-9 scores were significantly (p < 0.05) reduced in participants who came for the follow-up. They also showed significant (p < 0.01) increase in the score for all the domains of quality of life assessed by WHOQOL-BREF. 

**Conclusion: **The results indicate that motivational interviewing with art and music therapy can help in reducing stress, anxiety and depression scores and improve quality of life in patients who remains motivated and attend the follow up.

### P15 Supporting a national effort to implement alcohol use screening, brief interventions (SBI), medication-assisted therapy (MAT) in primary care

#### Tracy McPherson*

##### Public Health Department, NORC at the University of Chicago, Chicago, USA

###### Correspondence: Tracy McPherson (Mcpherson-tracy@norc.org)

*Addiction Science & Clinical Practice* 2022,**17(Suppl 1)**: P15

**Background: **In 2019, the Agency for Healthcare Research and Quality (AHRQ) launched a 4-year initiative to promote management of unhealthy alcohol use (UAU) in primary care. AHRQ contracted with NORC to create a Resource Center and conduct an evaluation across six grantees implementing alcohol screening, brief intervention (SBI), medication-assisted therapy (MAT) and/or referral to treatment in 750 primary care practices. The Resource Center's primary purpose is to support grantees in addressing UAU in primary care. To do so, it provides a centralized hub to collect tools, offers a robust environmental scan (e-scan) and an active Learning Community. A technical expert panel (TEP) provides guidance, resources, and co-facilitates workgroups. This initiative created an opportunity to evaluate best practices in supporting primary care practices implementing SBI and MAT during a public health crisis when changing priorities can overwhelm workflows.

**Methods: **The e-scan team reviewed over 2,000 source documents gathered through targeted literature reviews and working with AHRQ, grantees, TEP and partners. The Learning Community hosts monthly workgroups focused on practice facilitation, health information technology, and evaluation to provide a supportive venue for cross-grantee collaboration. Workgroups served as mini-communities for shared learning around topics like practice recruitment, practice facilitator responsibilities, and data collection. Over 125 stakeholders participated across 9 months. 

**Results: **Approximately 350 e-scan resources were identified and hosted on AHRQ's Academy, a national resource and coordination center. Lessons learned from the recruitment-focused workgroups included using warm handoffs, leveraging existing relationships, acknowledging effects of COVID-19, meeting practices where they are, and utilizing technology to support training/implementation. Identifying SBIRT champions that understand the practice culture was integral to strong implementation and data collection. 

**Conclusion: **Even amidst a pandemic, the project design enabled the UAU initiative to deliver assistance and resources that were responsive and relevant to grantees, practices, and the broader primary care community.

### P16 The RESPEKT campaign—does it work? Effects of a multi component mass media campaign to increase treatment seeking and reduce stigma of alcohol dependence

#### Sara W. Finn^1^, Anette S. Nielsen^2^

##### ^1^University of Southern Denmark and Karolinska Institutet, Sweden; Unit of Clinical Alcohol Research, Clinical Institute & Psychiatric Hospital, Region of Southern Denmark, University of Southern Denmark, Odense, Sweden

*Addiction Science & Clinical Practice* 2022,**17(Suppl 1)**: P16

**Background: **A minority of all with alcohol dependence seek treatment, where stigma is an important barrier to treatment seeking. In Denmark, a media campaign, "RESPEKT", has been broadcasted nationwide since 2015. The campaign is unique from an international perspective and aims to increase treatment seeking. Similar interventions have up until now not been scientifically evaluated. The aim of this study is to investigate campaign awareness, attitudes and stigma.

**Method: **Study design: Repeated cross-sectional study Participants: Adults aged 30 years and older in the general Danish population, n = 9000. Data: Pre and post the campaign, an online questionnaire was administrated by a market research company. This is a repeated survey that has been completed annually pre and post the campaign period between year 2017 and 2020. The questionnaire covered demographic data, alcohol use, campaign awareness and attitudes. Questions on stigma was added 2020. 

**Results: **Preliminary analyses, from 2017 to 2019, show that awareness of the campaign was high (60–78%). A majority (66–78%) described the campaign as "interesting", "trustworthy" and "relevant". The main message about free treatment was perceived by 22–40%. Support for offering free treatment increased from 83 to 89% between 2017 and 2019. Preliminary results from 2020 comparing pre to post campaign, show a small increase in stigma (mean difference 1.15, SD 0.42, p = 0.01). 

**Conclusion: **Adults in the general Danish population show high awareness of the campaign and express support for it and the main message. However, no effect on stigma is seen.

## Symposium Abstracts

### S1 A qualitative study to assess expectations, experience and benefits among substance users of a helpline based brief intervention

#### Ayushi Dubey

##### GSMC Mumbai, Mumbai, India

*Addiction Science & Clinical Practice* 2022,**17(Suppl 1)**: S1

**Background: **According to Centre for Disease Control and Prevention, about 33% of cardiovascular diseases related deaths and 40% of all cases of cancer are related to tobacco use. Patients often need help in taking necessary measures to quit tobacco use and it is important that adequate support is available and provided to enable them to make the change. Aim is to study the subjective experience of those who quit substances using quitline 'Mukti', that was run by medical students.

**Methods: **This study has a descriptive design and qualitative content analysis was used as the method. Participants who called Mukti quit helpline were included. Participants who were English speaking and substance free or reduced their intake in last 3 months were included, a total of five participants were interviewed and all were tobacco users.

**Results: **Among the following callers 3 tried past quit attempts but were not successful. Among expectations, three people seemed that their expectations were fully met. All five mentioned behavioural changes that changed after quitting or due to counselling. Three noticed change in interpersonal relationship especially with family members. All five had a better self-understanding and insight into their weaknesses and reason for addiction. Professional impact was noticed by two participants. And all five were able to noticed a change in knowledge about substances. Four were keen to encourage others to reduce their substance use. Example: I know that being a medical student I know it's harmful, but it's very hard to get from to get over it like it's very hard to quit it now. So I have to find a way to like completely quit it.

**Conclusion: **Participants found the support of helpline useful in quitting and experienced a significant improvement in quality of life after quitting tobacco. However, it is not known whether discussions would be experienced in the same way if patients who were less motivated, engaged.

### S2 An exploration of the feasibility and acceptability of delivering screening and brief interventions to women in prison

#### Jennifer Ferguson*, Aisha Holloway

##### Teesside University, Middlesbrough, UK

###### Correspondence: Jennifer Ferguson (Jennifer.ferguson@tees.ac.uk)

*Addiction Science & Clinical Practice* 2022,**17(Suppl 1)**: S2

**Background: **Whilst it is well evidenced that the prevalence of alcohol misuse is high in the criminal justice system, and it can be shown it is for women on their own also, it is important to investigate the differences between men and women, in order to tailor interventions to this specific population. More females are found to be risky drinkers when they arrive in prison (24%) compared to males (18%). An alcohol brief intervention (ABIs) is a secondary preventive activity aimed at those drinking in a way that is harmful to their health or wellbeing. Whilst ABI is well evidenced in primary care, there is a dearth of evidence in the criminal justice system, particularly with women in prison. This research aimed to approach this unmet need, to establish the feasibility and acceptability of ABI in this population.

**Methods: **12 in depth qualitative interviews were completed with residents in an open prison and 6 with relevant staff and stakeholders. Thematic analysis of the transcripts was undertaken and recommendations for a future pilot study were made.

**Results: **The research highlighted the importance of using the 10 question AUDIT to establish rapport as well as its main purpose of screening. Participants discussed pragmatic issues such as follow up in this vulnerable population, timing of the intervention components and the visual aid used to guide the intervention itself. One of the main findings was the element of staff rapport within the setting. It was surprisingly a uniformed officer who was most favoured for delivery of the intervention. The findings aligned with the already evidenced pains of imprisonment.

**Conclusions: **SBI with women in an open prison setting is both feasible and acceptable provided time the results of this study are implemented in the delivery.

### S3 Changes in substance use and depressive symptoms in youth during the COVID-19 pandemic

#### Verena E. Metz

##### Kaiser Permanente Division of Research, Oakland, CA, USA

*Addiction Science & Clinical Practice* 2022,**17(Suppl 1)**: S3

**Background: **Individuals globally were affected by the pandemic in myriad ways, including social isolation, and economic hardship, resulting negative impacts on mental health and substance use among many adults. Young people have been subjected to extraordinary changes as well but have received limited attention from research.

**Methods: **We used electronic health record data from a large U.S. healthcare system to compare self-reported substance use and depressive symptoms in youth (13–18 years) (N = 178,255) collected at annual pediatric well visits, pre- (3/1/2019–12/31/2019) and post-pandemic onset (3/1/2020–12/31/2020). We also examined self-reported alcohol use and alcohol problems (i.e. unhealthy drinking and alcohol use disorders (AUDs), N = 140,871) among young adult patients (18–34 years) during the same period.

**Results: **Compared to the year prior to the pandemic, after pandemic onset, significantly more adolescents reported alcohol use (7.75 vs. 7.42%, p = 0.02), depressive symptoms (30.03 vs. 24.06%, p < 0.0001) and suicidality (1.76 vs. 1.30%, p < 0.0001), whereas marijuana use decreased slightly (6.20 vs. 6.45%, p = 0.04). Young adults showed an initial drop in unhealthy drinking post pandemic onset, followed by a marked increase in the late summer/early fall of 2020, which peaked in September 2020 (653.7 vs. 521.6 cases of alcohol problems, and 491.2 vs. 358.7 cases of unhealthy drinking per 100,000 members, respectively, compared to September 2019, both p < 0.001). No significant differences were observed in AUD diagnoses.

**Conclusions: **Adolescents reported increased alcohol use and feelings of sadness and suicidality, perhaps related to less contact with peers, whereas young adults initially showed a dip in drinking, followed by a sharp increase—peaking about 6 months into the pandemic—compared to the previous year. Findings suggest considerable behavioral health burden among youth, resulting from the pandemic, and the imminent need for youth-serving systems to prepare to address these concerns as people begin to seek care.

### S4 Classifying user preferences and experiences with a no-code digital health application authoring tool

#### Steven J. Ondersma^1^, Brianna Broderick^2^, Allison Spiller^2^, Reema Kadri^3^, Gabriella Marcu^2^, Laurie Buis^3^

##### ^1^Michigan State University Division of Public Health, East Lasing, USA; ^2^University of Michigan School of Information, East Lasing, USA; ^3^University of Michigan Department of Family Medicine, East Lasing, USA

*Addiction Science & Clinical Practice* 2022,**17(Suppl 1)**: S4

**Background: **Scientific evaluation of digital applications for health behaviors is significantly hindered by obstacles related to cost, the need for access to significant technical expertise, the time it takes to obtain funding and then develop applications, and limitations in sharing and editing existing apps. Together, these obstacles slow the process of discovery, impede collaboration, and inhibit innovation. We interviewed researchers (none of whom had coding skills) using an intensive user-centered design framework in order to identify specific preferences and expectations, and to use that input as a guide to development of an open-source, non-commercial, no-code digital health app authoring platform.

**Methods: **A total of 29 researchers completed 32 interviews, 18 of which were contextual user interviews prior to software development, and 14 of which were usability tests of the beta authoring tool prototype. Participants identified their needs, preferences, and challenges in digital health app development, as well as their responses to potential/actual user interfaces. Interview recordings were transcribed and analyzed thematically in two rounds using memoing throughout.

**Results: **Participants overall expressed significant enthusiasm for the concept of digital health app authoring. Specific themes included a desire for "plug and play" content that can be modified, rather than always needing to develop content from scratch; a desire for flexibility with regard to text size, font, color, narrator, and overall appearance; the need for easy remote access even without research staff present; and for features allowing significant flexibility without sacrificing ease of use.

**Conclusions: **The enthusiasm expressed by participants for no-code digital health app authoring suggests that there is significant unmet need for such technology. This effort faces the challenge of balancing users' needs for power as well as ease of use.

### S5 Covid-19 pandemic and patterns of alcohol use among youths in Bayelsa State: SBIRT training commenced

#### Chia Francis

##### National Drug Law Enforcement Agency(NDLEA), Nigeria, West Africa

*Addiction Science & Clinical Practice* 2022,**17(Suppl 1)**: S5

**Background: **The first case of COVID-19 in Nigeria was declared on 27 February 2020. Today, the disease has spread to all parts of the country with reported cases of infections put at 163,330 and number of deaths pegged at 2,058(Daily Post, 2021). In Nigeria, the myth that alcohol has properties to kill the virus and the need to deal with a sense of loneliness and depression was associated with increasing youth alcohol use. This study therefore examined local myths about alcohol in relation to covid-19 prevention in Bayelsa state. The study also assessed the socio-economic and psychological impacst of alcohol consumption on youths as well as explored the challenges and solutions to addressing the above impacts among youths.

**Method: **This cross-sectional study employed two hundred and seventy-eight (278) youths as study participants. Instrument for data collection was the researcher`s structured questionnaire tagged: Covid-19 pandemic and current patterns of alcohol use questionnaire(C-19PCPAUQ). Research questions were analyzed using the simple mean (x), frequencies/percentages (%) and standard deviation (SD).

**Result: **Findings from this study showed that there is a local belief that alcohol can kill the Corona virus. For this reason, more youths were gulping alcohol. It was also found that lack of social activity, loss of job and depression resulting from covid-19 lockdown were also associated with increased alcohol use. Despite this, SBIRT services do not exist in health care centers in the state.

**Conclusion: **The results of this study are of great value as they informed the decision for the organization of the first SBIRT training for health workers recently in Bayelsa state. The overall opinion of the participants was that the implementation of SBIRT in primary health care centers is key to reducing the level of alcohol use and its impact on youths during and after the covid-19 pandemic.

### S6 COVID-19 related behavioural changes among young substance users

#### Sidharth Arya

##### State Drug Dependence Treatment Centre, Institute of Mental Health, Pt. BDS University of Health Sciences, Rohtak, India

*Addiction Science & Clinical Practice* 2022,**17(Suppl 1)**: S6

**Background: **People who use drugs are at increased risk of being exposed to COVID-19 as well as suffer from poorer outcomes. However, there is limited literature on the risky behaviours of young substance users which may put them at increased risk of COVID-19 infection.

**Methods: **We evaluated young individuals seeking treatment for substance use related issues after the lifting of the lockdown in India using a pre-tested structured questionnaire. The evaluation explored different substance related behaviours during the lockdown period including changes in substance use and related behaviours and adherence to universal safety precautions.

**Results: **Eighty-one male participants were enrolled, with 2/3rd of them using opioids. One-third reported increasing frequency and amount of substance respectively as coping strategy against COVID-19 pandemic. Sixty eight percent reported engagement risky behaviours with traveling (47%), wandering in groups (20%) and sitting in groups (17%) being most common. Universal safety precautions like wearing mask (95%) and handwashing (75%) were observed by majority, while home confinement and social distancing were observed by minority. Opioid users were less likely to adhere to stay home instructions (chi sq = 7.386, p = 0.016).

**Conclusion: **Our study points out that young substance users are prone to risky behaviours which may put them at higher risk of COVID-19 exposure. Brief interventions aimed at improving the knowledge and practice of universal safety behaviours can be a useful strategy in mitigating such exposures.

### S7 Effectiveness of brief alcohol interventions for pregnant women: Systematic literature review and meta-analysis

#### Svetlana Popova, Ekta Pandya, Danijela Dozet, Jurgen Rehm

##### Institute for Mental Health Policy Research & Campbell Family Mental Health Research Institute, Centre for Addiction and Mental Health, (CAMH), University of Toronto, Toronto, Canada

*Addiction Science & Clinical Practice* 2022,**17(Suppl 1)**: S7

**Background: **Prenatal alcohol exposure (PAE) can result in a range of adverse neonatal outcomes, including Fetal Alcohol Spectrum Disorder (FASD). This study aimed to investigate the effectiveness of brief interventions (BIs) in eliminating or reducing 1) alcohol consumption during pregnancy; and 2) PAE related adverse neonatal outcomes.

**Method: **A comprehensive systematic literature search was conducted for original quantitative studies (randomized control trials (RCTs); quasi-experimental) in any setting, published from 1987 to 2021. The comparison group was no/minimal intervention, where a measure of alcohol consumption was reported. Studies were critically appraised using Centre for Evidence-based Medicine Oxford critical appraisal tool for RCT. Meta-analysis of continuous and binary estimates of effect-size for similar outcome measures for BIs versus control groups were pooled and reported as Cohens' D and odds ratios (ORs), respectively. 

**Results: **In total, 26 studies from high-income countries compared BIs to control groups: 18 RCTs, 4 C-RCTs, 4 experimental studies (non-equivalent groups). The meta-analysis included 22 studies, which randomized a total of 4,865 participants. The pooled estimates of percentage of abstinence during pregnancy in 12 BI versus control arms (n = 2,620) show BI group had 56% higher odds at post-test of being abstinent compared to control (OR = 1.56, 95% CI = 1.15–2.13, I2 = 46.75%). No statistically significant difference was observed for mean drinks/week and AUDIT scores in BI versus control groups at post-test. Seven studies reported neonatal outcomes. Meta-analysis of 3 BI versus control arms (n = 740) observed 33% lower odds of preterm birth compared to the control (OR = 0.67, 95% CI = 0.46–0.98, I2 = 0.00%). No statistically significant difference observed for Apgar score, mean birthweight, and low-birth weight. 

**Conclusion: **BIs are moderately effective in increasing abstinence during pregnancy and preventing preterm births. More studies on the effectiveness of BIs are needed from low- and middle-income countries, as well as with younger mothers, and ethnic groups.

### S8 Harnessing social media and starting of a helpline by medical students to help people decrease substance use and create an environment that facilitates substance free lifestyle.

#### Ojas Krishnani

##### Medical student, GSMC Mumbai, Mumbai, India

*Addiction Science & Clinical Practice* 2022,**17(Suppl 1)**: S8

**Background: **Smoking and alcohol are amongst the top 5 risk factors for early death and disability. Per capita consumption of alcohol in India increased by 55 percent from 1992–2012. Students are a huge population of determined individuals who can make a difference, however there is a paucity of data regarding their impact on this issue.

**Methods: **A model called "Mukti" was created which is a group of medical and other students in June, 2020, which aims to—a) make people aware of the evidence about substance use, b) empower people and their family members to overcome the challenge of substance use, c) sensitise medical students to this problem and increase their skills to support people in quitting, and d) cultivate an environment on campuses that facilitates substance free lifestyle. It was done by—1) creating awareness through posters and videos on social media, 2) on-call free helpline operated by medical students trained and supervised by psychiatrists. They were trained in evidence based-practices like Brief intervention, motivational interviewing, relapse prevention, craving management, supportive counselling of relatives, and 3) collaboration with youth groups to create awareness on substance use.

**Results: **On social media, the reach was 55,744 through 73 posts. There are a total of 140 volunteers in Mukti. 21 volunteers either decreased/contemplated about their substance use. Out of a total of 19 people who called on helpline, 9 people are abstinent for more than 3 months and 3 more have decreased it significantly. There were 2 training sessions conducted, each being of 20 h attended by 28 students. 3 ongoing research projects. Collaborated with 5 organisations.

**Conclusion: **The interventions of the model have had a positive impact on volunteer empowerment, creating awareness and people who called on the helpline.

### S9 Impact of a short role-play based online training of Medical students on their Motivational Interviewing skills

#### Aditya Shah^1^, Divya Shrinivas^2^, Daniyaal Taheri^3^, Nikita Chandak^3^, Aarya Desai^3^, Sachi, Anoop Bharti, Rohit Ganorkar

##### ^1^GSMC Mumbai, Maharashtra, India; ^2^GMC Ambajogai, Maharashtra, India; ^3^SSMC Mumbai, Maharashtra, India

*Addiction Science & Clinical Practice* 2022,**17(Suppl 1)**: S9

**Background: **Substance use is one of the leading causes of mortality worldwide. Motivational Interviewing (MI) has known to be an important counselling method to promote behavioral changes like quitting substance use or change in diet or exercising. Hence, training medical and paramedical students in this skill has become crucial. The aim is to evaluate the impact of a role play based online training in MI on students who had volunteered for a quitting helpline, 'Mukti'.

**Methods: **Medical students underwent a voluntary online video conference-based training in MI. The training was given by a group of psychiatrists and was part of a broader 24 h-training on substance cessation skills, conducted after college hours and on weekends, spread over 7 days. The module on MI was for 2 h. The skill assessment was done through a pre-validated tool, Video Assessment of Simulated Encounter- Revised (VASE-R) 1 month after the training. 10 students enrolled in the control group (CG) and study group (SG) each. Data was analyzed using t-test.

**Results: **The average total score of MI assessment in the interventional and control groups were 20.8 and 11 respectively (P = 0.03). The average score in each domain comparing the two groups (SG vs CG) were as follows: Reflective listening (4.2 vs 2.4), responding to resistance (5.8 vs 3.1), summaries (3.3 vs 1.9), developing discrepancy (4.2 vs 2.1) and eliciting change talk (3.3 vs 1.5).

**Conclusion:** Medical students have poor awareness about MI skills even though it has become important counselling method in today's era of lifestyle diseases. A short training with sufficient role plays can enhance MI skills of medical students significantly enabling them to provide better counselling to people with substance use disorders.

### S10 Pregnancy without alcohol or drugs: a multicomponent model implementation

#### Lidia Segura, Ana Ibar, Carla Bruguera, Joan Colom

##### ASPCAT, Barcelona, Spain

*Addiction Science & Clinical Practice* 2022,**17(Suppl 1)**: S10

**Background: **"Pregnancy without alcohol or drugs" aims to raise awareness of the risks of pregnancies exposed to alcohol and other substances and to widely implement SBIRT strategies. It was developed by the Programme on Substance Abuse of the Public Health Agency of Catalonia in the framework of the "Pregnancy monitoring protocol" and entails multilevel implementation strategies.

**Methods: **The programme promotes the creation of perinatal mental health circuits, i.e. local multi-professional networks including mental health services, reproductive health centres, specialised addiction treatment centres and hospitals. Along with training, AUDIT and ASSIST were introduced in the electronic health records facilitating preventive activities. Finally, the programme also creates materials to raise awareness among child-bearing age women including posters, videos and other informational resources. Evaluation of the implementation includes among others, the analysis of screening and intervention rates in sexual and reproductive services and the follow-up of the work of the perinatal mental health circuits.

**Results: **Across the region, 10 trainings took place involving a variety of professional profiles such as midwives, nurses, obstetricians, addiction and mental health specialists. Over 60% of trainees approved the content structure, valued the materials provided and would recommend the training to another professional. 10 perinatal mental health circuits across the region have been created involving mental health and addiction treatment services, hospitals and reproductive health centres. 43 reproductive health centres have used AUDIT as screening tool and up to 50% of women attending some of these centres have been screened for their alcohol consumption during pregnancy.

**Conclusion: **Addressing exposed pregnancies requires multi-component inter-departmental supportive actions that are respectful to women autonomy and that take into account the wide variety of alcohol and other substances use determinants. The commitment and collaboration of all public health and social services are important to reduce the risks for the mother and the baby. 

### S11 Screening and Brief Intervention for the reduction of alcohol and drug exposed pregnancies—the rationale and the objectives for the launch of a new INEBRIA special Interest Group

#### Lidia Segura, Lela R. McKnight-Eily^2^, Svetlana Popova^3, 4^

##### ^1^ASPCAT, Barcelona, Spain; ^2^U.S. Centers for Disease Control and Prevention National Centers on Birth Defects and Developmental Disabilities, Atlanta, USA; ^3^Institute for Mental Health Policy Research & Campbell Family Mental Health Research Institute, Centre for Addiction and Mental Health, (CAMH), Toronto, Canada; ^4^University of Toronto, Toronto, Canada

*Addiction Science & Clinical Practice* 2022,**17(Suppl 1)**: S11

**Background: **The association between prenatal alcohol consumption (particularly frequent binge drinking) and fetal alcohol syndrome in persons exposed prenatally is well-established, although the causal factors include a complex algorithm that may vary by individual with many unknown factors. Less is known about the impact of prenatal exposure of other legal and illegal drugs, like opioids, on a developing baby. Despite the growing literature on the topic, no safe substance consumption limits have been defined and therefore the public health recommendation is to promote alcohol and drug-free pregnancies. However, there are still a number of questions around prevention including the role that SBI may play. In the context of INEBRIA, there has been a growing interest in this area. The leads of the SIG-Substance-Free Pregnancies would like to update the work done and the future plans.

**Methods: **First, we will review the overall themes from the last meeting (the goal of the SIG, the role of partners in preventing AEPs) and next steps from the INEBRIA 2019 SIG meeting (working on the development of a consensus statement for the SIG to submit for publication, worldwide literature and qualitative interview review of best practices in the prevention of alcohol exposed pregnancies) to discuss any unresolved issues (language barriers, technology platform for quarterly meetings). We will update the group on the progress that the SIG has made to date (proposal of a symposium for the 2020 conference, consideration of SIG products). Then, we will engage in open discussion with the goal of mobilizing members towards the further creation of the SIG by agreeing upon the aims, organization and activities that could be undertaken, especially in the promotion of collaborative research options on this important topic. We will also discuss the evidence and gaps. Finally, we will use time to finalize the SIG consensus statement.

### S12 The Alcohol e-Help study: Methodology of the multicentre randomized controlled trial

#### Michael P. Schaub

##### Swiss Research Institute for Public Health and Addiction (ISGF), University of Zurich, Zurich, Switzerland

*Addiction Science & Clinical Practice* 2022,**17(Suppl 1)**: S12

**Background: **Given the scarcity of alcohol prevention and alcohol use disorder treatments in many low and middle-income countries, the World Health Organization (WHO) launched an e-health portal on alcohol and health that includes a Web-based self-help program. A multicentre randomized controlled trial (RCT) was conducted to test the efficacy of the internet-based self-help intervention to reduce alcohol use in four countries (Belarus, Brazil, India & Mexico).

**Methods: **Two-arm randomized controlled trial (RCT) with follow-up 6 months after randomization.

Setting: Community samples in middle-income countries.

Participants: People aged 18+, with Alcohol Use Disorders Identification Test (AUDIT) scores of 8+ indicating hazardous alcohol consumption.

Intervention and control groups: Offer of an internet-based self-help intervention, 'Alcohol e-Health', was compared with a psycho-educative 'waiting list' control group. The intervention, adapted from a previous program with evidence of effectiveness in a high-income country, consisted modules to reduce or entirely stop drinking. Measurements: The primary outcome measure is change in the Alcohol Use Disorders Identification Test (AUDIT) score assessed at 6-month follow-up. Secondary outcomes include self-reported the numbers of standard drinks and alcohol-free days in a typical week during the past 6 months, and cessation of harmful or hazardous drinking (AUDIT < 8). Intention treat analysis was conducted to ascertain changes in AUDIT score in intervention compared to the control group.

**Trial Registration: **ISRCTN14037475

### S13 The effectiveness of the World Health Organization (WHO) Internet self-help intervention “Alcohol e-Help” for the reduction of alcohol consumption in India: A randomized controlled trial

#### Roshan Bhad

##### National Drug Dependence Treatment Centre (NDDTC) & Department of Psychiatry, All India Institute of Medical Sciences (AIIMS), New Delhi, India

*Addiction Science & Clinical Practice* 2022,**17(Suppl 1)**: S13

**Background: **India suffers from a significant treatment gap for alcohol use disorders largely due to limited availability of trained professionals. Web based interventions are promising due to their better accessibility, low threshold for help-seeking, relative anonymity, property of empowering the clients (guided self-help), and the low costs.

**Methods: **As a part of multi country WHO project (Belarus, Brazil, India & Mexico), a web-based self-help intervention “Alcohol e-Help”, was made available through the India-specific portal https://www.alcoholwebindia.in/, in English language. The Indian arm of the RCT followed the same protocol as elsewhere and clearance from Ethics Committee of AIIMS, Delhi was secured. Participant recruitment strategies included putting up posters and flyers in health settings and newspaper advertisements.

**Results: **In India, 217 participants registered in the RCT, intervention group (n = 95), control group (n = 117), 5 participants did not fulfil inclusion criteria. Mean age of participants in intervention group was 38.6 (SD 9.5) years and control group was 40.3 (SD 8.9) years. Mean baseline AUDIT score was 30.2 in both groups. Follow up rate (6 months) was 49% in both groups. Mean AUDIT change at the end of follow up was 7.5 (SD = 5.7) and 4.6 (SD = 8.2) in intervention and control group respectively which was statistically significant in the intention to treat analysis (B = − 2.89, 95% CI − 5.61 to − 0.18, P = 0.037, d = 0.41).

**Conclusions: **The web-based intervention for alcohol use disorders appeared a feasible, acceptable and an effective approach likely to be useful for addressing the treatment gap in India. Future research should address developing and evaluating interventions which are accessible through mobile devices and in Indian languages.

### S14 The effectiveness of the World Health Organization (WHO) Internet self-help intervention “Alcohol e-Help” for the reduction of alcohol consumption in Brazil: A randomized controlled trial

#### Maria L. O. Souza‐Formigoni*, André L. M. Andrade, Fabricio L. Moraes

##### Departamento de Psicobiologia, Escola Paulista de Medicina, Universidad Federal de São Paulo, São Paulo, Brazil

###### Correspondence: Maria L. O. Souza‐Formigoni (mlosformigoni@unifesp.br)

*Addiction Science & Clinical Practice* 2022,**17(Suppl 1)**: S14

**Background: **There is a significant gap in treatment of alcohol use disorders due to limited trained professionals. Web-based interventions with their increased accessibility, low threshold for help-seeking, relative anonymity, emphasis on patients' active role in (guided) self-help, and their low costs can minimize the treatment gap.

**Methods:**As a part of the multi country WHO project (Belarus, Brazil, India & Mexico), a web-based self-help intervention “Alcohol e-Help”, available round-the-clock for 6 weeks, was developed. After pilot testing, efficacy of the “Alcohol e-Help” intervention in comparison to a psycho-educative information controlled group was investigated using a randomized controlled trial design (RCT). Brazil had a separate website (https://www.informalcool.org.br/bebermenos) tailored to address the needs of Brazilian population. Data were collected and analysed as per study protocol. Recruitment strategies included notes in print media, universities websites, television and radio coverage.

**Results:**In Brazil, 587 participants registered in the RCT, 290 in the intervention group and 297 in the control group. Mean age of participants in the intervention group was 37.6 (SD = 10.6) years and in the control group 36.6 (SD = 10.2) years. Mean baseline AUDIT score was 22.2 in both the groups. Follow-up rate (6 months) was 28% (intervention group) and 38% (control group). Mean AUDIT change at the end of follow-up was 5.1 (SD = 8.4) and 1.8 (SD = 6.6) in intervention and control group, respectively, which was statistically significant in the intention to treat analysis (B = − 3.23, 95% CI − 5.42 to − 1.03, P = 0.004, d = 0.43).

**Conclusions: **Web-based intervention for alcohol use disorders is feasible and acceptable. It is likely to be useful for the treatment of low/medium level of alcohol dependence in the Brazilian population.

**Trial registration: **ISRCTN14037475, https://doi.org/10.1186/ISRCTN14037475. International Registered Report Identifier (IRRID): RR2-10.1111/add.14034

### S15 The effectiveness of the World Health Organization (WHO) Internet self-help intervention “Alcohol e-Help” for the reduction of alcohol consumption in Mexico: A randomized controlled trial

#### Marcela A. T. Sainz

##### National Institute of Psychiatry, Department of Social Sciences in Health, Direction of Epidemiological and Psychosocial Research, Mexico City, Mexico

*Addiction Science & Clinical Practice* 2022,**17(Suppl 1)**: S15

**Background: **There is a significant gap in treatment of alcohol use disorders due to limited trained professionals. Web-based interventions with their increased accessibility, low threshold for help-seeking, relative anonymity, emphasis on patients' active role in (guided) self-help, and their low costs can minimize the treatment gap.

**Methods: **As a part of the multi country WHO project (Belarus, Brazil, India & Mexico), a web-based self-help intervention “Alcohol e-Help”, available round-the-clock for 6 weeks, was developed. After pilot testing, efficacy of the “Alcohol e-Help” intervention compared to a psycho-educative information control group was investigated using a randomized controlled trial design (RCT). Mexico had a separate website tailored to address the needs of Mexican population (https://www.saberdealcohol.org.mx). Data were collected and analysed as per study protocol. Recruitment strategies included notes in print media, information flyers, social media, television and radio coverage.

**Results: **In Mexico, 491 participants registered in the RCT, 246 in the intervention group and 245 in the control group. Mean age of participants in the intervention group was 36.6 (SD = 11.0) years and in the control group 36.6 (SD = 10.6) years. Mean baseline AUDIT score was 22.5 and 22.2 in intervention and control groups respectively. Follow-up rate (6 months) was 33% (intervention group) and 57% (control group). Mean AUDIT change at the six months follow-up was 9.6 (SD = 7.5) and 3.4 (SD = 7.2) in intervention and control group, respectively, which was statistically significant in the intention to treat analysis (B = − 6.16, 95% CI − 8.23 to − 4.09, P =  < 0.001, d = 0.84).

**Conclusions: **Web-based intervention for alcohol use disorders is feasible and acceptable. It is likely to be useful for the treatment of low/medium level of alcohol dependence in the Mexican population.

### S16 The impact on young people will still be a shadow a lot further down the line: Examining the costs of the pandemic for young people

#### Andrew Divers

##### Teesside University, Middlesbrough, UK

*Addiction Science & Clinical Practice* 2022,**17(Suppl 1)**: S16

**Background:**The impact of COVID has been felt by everyone. Yet, as the pandemic has dragged on, it has become more and more clear that there are those for whom the exacerbation of existing inequalities, or the increased upheaval and uncertainty of their lives means that although we have all been touched by pandemic, we are not ‘all in this together'.

**Methods:**This paper will introduce the experiences of 12 young people (aged 18–25) from a wider study of 52 adults in the Middlesbrough area of the UK during the pandemic, exploring issues including mental health, wellbeing and drug and alcohol taking behaviour (and the risk factors for such behaviours). **Results:**Showed increased pressure of disruption to socialisation during one's essential ‘formative years', the worries over wholesale changes to education—which have themselves widened pre-existing inequalities of access and achievement—the unknown impact of the pandemic on the future, and the added stigma of blame for spikes in infection have all contributed to a significant decrease in the health and wellbeing of young people. Interviewees spoke of changes to the way in which they socialised and connected with others, and how this in turn affected the way in which they used alcohol and drugs during this time. "Alcoholism and drug taking has skyrocketed during this lockdown, you see if people were allowed to go out and about and socialise with other people, then maybe they wouldn't be taking so many drugs, maybe they wouldn't be drinking the odd case [of beer]."

**Conclusions:**As we start to exit from the pandemic lockdown more work is needed to develop brief interventions for young people to tackle the multiple health and social needs they have encountered.

### S17 To evaluate the effectiveness of a brief intervention for substance use offered by medical students through a quit line

#### Divya Shrinivas^1^, Ojas Krishnani^2^, Dharav Shah

##### ^1^GMC Ambajogai, Maharashtra, India; ^2^GSMC Mumbai, Maharashtra, India

*Addiction Science & Clinical Practice* 2022,**17(Suppl 1)**: S17

**Background: **About 55% of smokers are interested in quitting the habit or plan to do so, as per the GATS report. Similarly, users of most substances want to quit, but are unable to do so. Lockdown during pandemic brought a unique opportunity to quit, because of increased motivation as well as decreased availability. To respond to this need of the hour, we established a quit line for those wanting to quit their substance use.

**Methods: **Students volunteers wanting to participate underwent training about the harms associated with addictive substances and how to support people to quit. Training was undertaken on zoom platform by a group of psychiatrists. A helpline was established and popularised through social media platforms. psychoeducational posters and videos were also circulated to add to people's knowledge and build the motivation to quit. Calls received till 3 months back, were included in this analysis.

**Results: **In total 19 individuals called the helpline of which the mean age of the individuals was 32.8 years (18–55 years). All were males. Out of these n = 16 (84%) had called to reduce tobacco use (TU) and n = 3 (15.7%) had called to reduce alcohol use (AU). Additionally, 7 distressed family members of substance users also called who were seeking advice. 8 (42%) were perusing an educational degree and 11 (58%) were only working. Out of total TU, 6 (37.5%) were able to quit it, 3 (18.7%) were able to reduce its use and 7 (44.3%) were unable to reduce consumption within 3 months. Out of total AU 3, all were able to quit alcohol use over 3 months period. All those who quit or reduced substance (n = 12) noticed improvement in savings.

**Conclusion: **Helpline run by adequately trained medical students. can be an effective model to support people in quitting substance use.

### S18 What does the evidence tell us about the efficacy of ASBIs in the prison system?

#### Dorothy Newbury-Birch*, Jennifer Ferguson, Aisha Holloway

##### Teesside University, Middlesbrough, UK

###### Correspondence: Dorothy Newbury-Birch (d.newbury-Birch@tees.ac.uk)

*Addiction Science & Clinical Practice* 2022,**17(Suppl 1)**: S18

**Background: **Levels of risky drinking and dependency are high amongst incarcerated individuals. Despite very little evidence of ASBIs in the criminal justice system services are currently advocating the use of brief interventions in the criminal justice system. We carried out a systematic review the literature on the efficacy of ASBIs for incarcerated individuals to ascertain the efficacy or effectiveness in making changes to either consumption of alcohol or other social outcomes.

**Methods: **A systematic review of randomised controlled trials or matched group trials of the efficacy of psychosocial alcohol interventions for incarcerated individuals (adolescents and adults) was carried out. We searched seven databases, with no restrictions on language, year or location from inception through to August 2017. The Critical Appraisal Skills Programme tool was used to assess the quality of included studies. The Template for Intervention Description and Replication (TIDieR) checklist was used to ascertain intervention descriptions.

**Results: **Nine studies from 11 papers were included in the analysis. Eight of the studies were from the USA and one from the UK. Six of the studies included brief interventions and three extended interventions. Of the nine included studies three were low quality, five a medium risk and 1 high quality. Every study used a different measure of alcohol consumption. Three of the studies that looked at brief interventions and all of the three extended intervention studies found significant reductions in relation to alcohol outcomes. The TIDieR results showed that the brief interventions studies were all based on Miller and Rollnick and interventions lasted 30–60 min whilst the extended interventions lasted between 12 and 20 h.

**Conclusions:** Although there are some promising findings in relation to efficacy the numbers of studies and participants is too low. More research is needed in this setting to ascertain efficacy.

### S19 What is the learning from a pilot feasibility RCT of ASBI for male remand prisoners carried out during a pandemic?

#### Aisha Holloway^1^*, Victoria Guthrie^2^, Jamie Smith^2^, Jennifer Ferguson^3^, Dorothy Newbury-Birch^3^

##### ^1^University of Edinburgh, Edinburgh, UK; ^2^School of Health in Social Science, University of Edinburgh, Edinburgh, UK; ^3^Teesside University, Middlesbrough, UK

###### Correspondence: Aisha Holloway (aisha.holloway@ed.ac.uk)

*Addiction Science & Clinical Practice* 2022,**17(Suppl 1)**: S19

**Background: **Evidence tells us that intensive interventions that target high-risk offenders work best for reducing recidivism and this is where resources are being placed. However, services are currently advocating the use of brief interventions in the criminal justice system.

**Methods: **APPRAISE is using mixed methods, with two linked phases, across two remand prisons in the UK, recruiting 180 adult men on remand (90 Scotland, 90 England). Phase one is a two-arm, parallel-group, individually randomised pilot study with follow-ups with participants at six and 12 months. The pilot evaluation will be used to inform a future definitive multicentre RCT. Phase two is a process evaluation of the study which includes qualitative work with stakeholders and participants.

**Results: **Prior to the pandemic lockdown in the UK we had recruited all participants to the English arm of the study and half of the Scottish participants. As prisons were locked down we were unable to go back into the prison to recruit any more participants. Further issues were encountered when it came to tracking individuals as prison workers were so busy. This meant that other ways were needed to be found to do the work and we have conducted all our interviews online and will continue to do this. We have been granted an extension for the research to enable us to finish the study.

**Conclusions: **It has been possible to continue the study despite the pandemic however changes have had to be made which includes moving all interviews online. We will carry out a survey of all remand prisons in the UK to ascertain how alcohol interventions have been affected by the pandemic.

### S20 What is the prevalence of alcohol use disorders within the criminal justice system in the UK?

#### Dorothy Newbury-Birch*, Jennifer Ferguson, Aisha Holloway

##### Teesside University, Middlesbrough, UK

###### Correspondence: Dorothy Newbury-Birch (d.newbury-Birch@tees.ac.uk)

*Addiction Science & Clinical Practice* 2022,**17(Suppl 1)**: S20

**Background: **In the UK it has been shown that the prevalence rate of risky drinking (using the AUDIT tool and a score of 8+) is around 20–30% with around 4% being dependent in the general population. There is a wealth of evidence on the effectiveness of brief interventions in primary care however very little is known about the prevalence of risky drinking in the criminal justice system. It is imperative to know the prevalence rates to understand the interventions needed.

**Methods: **We carried out a systematic review of literature in 2015 to ascertain the prevalence rates of risky drinking in different stages of the criminal justice system in the UK (adults in the police custody, probation, prison and young people). We searched Pubmed, Scopus and Medline and only included studies that used the Alcohol Use Disorders Identification Test (AUDIT) to enable comparison across studies.

**Results: **Fourteen studies were included (five in the police custody setting, two in the probation setting, six in the prison setting and one with young people). The review found high levels of risky drinking; 64–88% in the police custody setting; 53–69% in the probation setting and 59–67% in the prison system. Furthermore, 64% of young people were risky drinkers (using adult cut-offs for AUDIT). There were high levels of dependency; 21–38% in the police custody setting; 17–35% in the probation setting and 8–43% in the prison setting. Furthermore, 39% of youths were categorized as dependent using adult cut-offs on AUDIT and 77% using youth cut-offs (2+).

**Conclusions: **There are much higher levels of risky and dependent drinking within the criminal justice system and more research is needed to ascertain whether brief interventions will work in this setting.

